# SHP2 Regulates Chondrocyte Terminal Differentiation, Growth Plate Architecture and Skeletal Cell Fates

**DOI:** 10.1371/journal.pgen.1004364

**Published:** 2014-05-29

**Authors:** Margot E. Bowen, Ugur M. Ayturk, Kyle C. Kurek, Wentian Yang, Matthew L. Warman

**Affiliations:** 1 Orthopaedic Research Laboratories, Boston Children's Hospital, Boston, Massachusetts, United States of America; 2 Department of Orthopaedics, Brown University, Providence, Rhode Island, United States of America; University of Oxford, United Kingdom

## Abstract

Loss of *PTPN11*/SHP2 in mice or in human metachondromatosis (MC) patients causes benign cartilage tumors on the bone surface (exostoses) and within bones (enchondromas). To elucidate the mechanisms underlying cartilage tumor formation, we investigated the role of SHP2 in the specification, maturation and organization of chondrocytes. Firstly, we studied chondrocyte maturation by performing RNA-seq on primary chondrocyte pellet cultures. We found that SHP2 depletion, or inhibition of the ERK1/2 pathway, delays the terminal differentiation of chondrocytes from the early-hypertrophic to the late-hypertrophic stage. Secondly, we studied chondrocyte maturation and organization in mice with a mosaic postnatal inactivation of *Ptpn11* in chondrocytes. We found that the vertebral growth plates of these mice have expanded domains of early-hypertrophic chondrocytes that have not yet terminally differentiated, and their enchondroma-like lesions arise from chondrocytes displaced from the growth plate due to a disruption in the organization of maturation and ossification zones. Furthermore, we observed that lesions from human MC patients also display disorganized chondrocyte maturation zones. Next, we found that inactivation of *Ptpn11* in *Fsp1-Cre*-expressing fibroblasts induces exostosis-like outgrowths, suggesting that loss of SHP2 in cells on the bone surface and at bone-ligament attachment sites induces ectopic chondrogenesis. Finally, we performed lineage tracing to show that exostoses and enchondromas in mice likely contain mixtures of wild-type and SHP2-deficient chondrocytes. Together, these data indicate that in patients with MC, who are heterozygous for inherited *PTPN11* loss-of-function mutations, second-hit mutations in *PTPN11* can induce enchondromas by disrupting the organization and delaying the terminal differentiation of growth plate chondrocytes, and can induce exostoses by causing ectopic chondrogenesis of cells on the bone surface. Furthermore, the data are consistent with paracrine signaling from SHP2-deficient cells causing SHP2-sufficient cells to be incorporated into the lesions.

## Author Summary

Patients with the inherited disorder, metachondromatosis (MC), develop multiple benign cartilage tumors during childhood. MC patients carry heterozygous loss-of-function mutations in the *PTPN11* gene, and their cartilage tumors likely arise when the second *PTPN11* allele is lost due to a somatic mutation. *PTPN11* encodes a phosphatase called SHP2 that is involved in a variety of signaling pathways. Here, we use mouse models and cell culture assays to investigate the mechanisms by which loss of SHP2 promotes cartilage tumor formation. We show that cartilage tumors that form inside bones (enchondromas) likely arise due to disorganized growth and delayed terminal differentiation of growth plate chondrocytes, while cartilage tumors that form on the bone surface (exostoses) can arise due to ectopic chondrogenesis of fibroblast-like cells that surround bones. We also suggest that paracrine signals from SHP2-deficient cells cause neighboring SHP2-sufficient cells to contribute to exostoses and enchondromas. Finally, we provide *in vitro* data that the ERK1/2 pathway is regulated by SHP2 and promotes chondrocyte terminal differentiation. Together, our data provide insight into the mechanisms underlying cartilage tumor formation and implicate SHP2 as a key regulator of chondrocyte specification, organization and maturation.

## Introduction

Enchondromas in bone cavities and exostoses on bone surfaces are the two most common types of benign cartilage tumors [1]. Both tumors occur in patients with the autosomal dominant heritable disorder metachondromatosis (MC) [2]. Families with MC segregate heterozygous loss-of-function mutations in *PTPN11,* and their cartilage tumors are thought to arise from cells that have second-hit somatic *PTPN11* mutations [2],[3]. *PTPN11* encodes the tyrosine phosphatase, SHP2, which has cell type-dependent and context-dependent roles in many signaling pathways [4]. MC is unique in that it is associated with both enchondromas and exostoses. Other genetic conditions cause enchondromas or exostoses, but not both. Patients with Ollier disease and Maffucci syndrome, due to somatic gain-of-function mutations in *IDH1* or *IDH2,* develop enchondromas [5]–[7]. Patients with heterozygous loss-of-function mutations in *EXT1* or *EXT2* segregate multiple hereditary exostoses (MHE) [8]. The mechanism by which cartilage tumors arise in patients with MC is incompletely understood. Also unknown is whether tumor formation in patients with MC results from perturbation of the same biologic pathway(s) that are affected in patients with Ollier disease, Maffucci syndrome, and MHE.

Mice have been used to model tumor formation caused by SHP2 deficiency. Unlike humans, cartilage tumors have not been observed in mice that are heterozygous for *Ptpn11* loss-of-function mutations, and complete absence of *Ptpn11* in mice causes embryonic lethality [9]. However, cartilage tumors can be induced by a conditional biallelic inactivation of *Ptpn11* [10]–[12], which mimics somatic second-hit *PTPN11* mutations that likely give rise to cartilage tumors in human MC patients. Mosaic inactivation of *Ptpn11* in chondrocytes and/or perichondrial cells produces enchondromas in the vertebrae and ribs, and exostoses in the limbs [11]–[13]. These data suggest that exostoses arise from *Ptpn11*-null perichondrial cells that inappropriately differentiate into chondrocytes [11],[12], and that enchondromas arise from *Ptpn11*-null growth plate chondrocytes that fail to be replaced by bone [12]. Thus, SHP2 appears to participate in the specification, proliferation, and maturation of chondrocytes.

One downstream signaling pathway likely altered in SHP2-deficient chondrocytes is the ERK1/2 pathway. Exostoses in *Ptpn11*-mutant mice have reduced levels of phospho-ERK1/2 [11],[12]. While *in vivo* studies have not directly demonstrated that inactivation of the ERK1/2 pathway is sufficient to produce cartilage tumors, studies have shown that inactivation of *Erk1* and *Erk2* in mesenchymal cells leads to ectopic cartilage clusters in the perichondrium of mouse embryos [14]. Therefore, it is possible that these ectopic clusters could develop into exostoses had the mice survived into adulthood. Furthermore, inactivation of *Erk1* and *Erk2* in chondrocytes disrupts the growth plate's columnar-organization, causes chondrocytes to hypertrophy prematurely, and delays the replacement of cartilage with bone [14],[15]. Thus, reduced ERK1/2 signaling following SHP2 depletion may mediate some of the phenotypic features in patients with MC.

Because the molecular consequences of SHP2 depletion or ERK1/2 inactivation in chondrocytes are incompletely understood, we investigated the transcriptional response to either SHP2 depletion or MEK1/2 inhibition in chondrocyte pellet cultures by performing massively parallel mRNA sequencing (RNA-seq). We also conditionally inactivated *Ptpn11 in vivo* and examined the effects of SHP2 depletion on chondrocyte maturation and specification, and we examined chondrocyte maturation in exostosis lesions from MC patients. Herein, we report that SHP2 and the ERK1/2 pathway have overlapping effects on gene expression during chondrocyte maturation in pellet culture. We suggest a mechanism by which enchondromas arise in MC patients that involves disrupted growth plate architecture and delayed terminal differentiation, and show that exostoses can arise following inappropriate chondrogenic differentiation of fibroblasts. We also propose that cartilage lesions contain a mixture of SHP2-deficient and normal cells. Lastly, the RNA-seq method and transcriptome database we developed should be useful to other scientists who are interested in growth plate biology and chondrocyte maturation.

## Results

### RNA-Seq Analysis Of Pellet Cultures Identifies Both Previously Known And Novel Transcriptional Changes That Occur As Chondrocytes Mature From The Proliferative Through To The Late-Hypertrophic Stage

**Figure 1:**
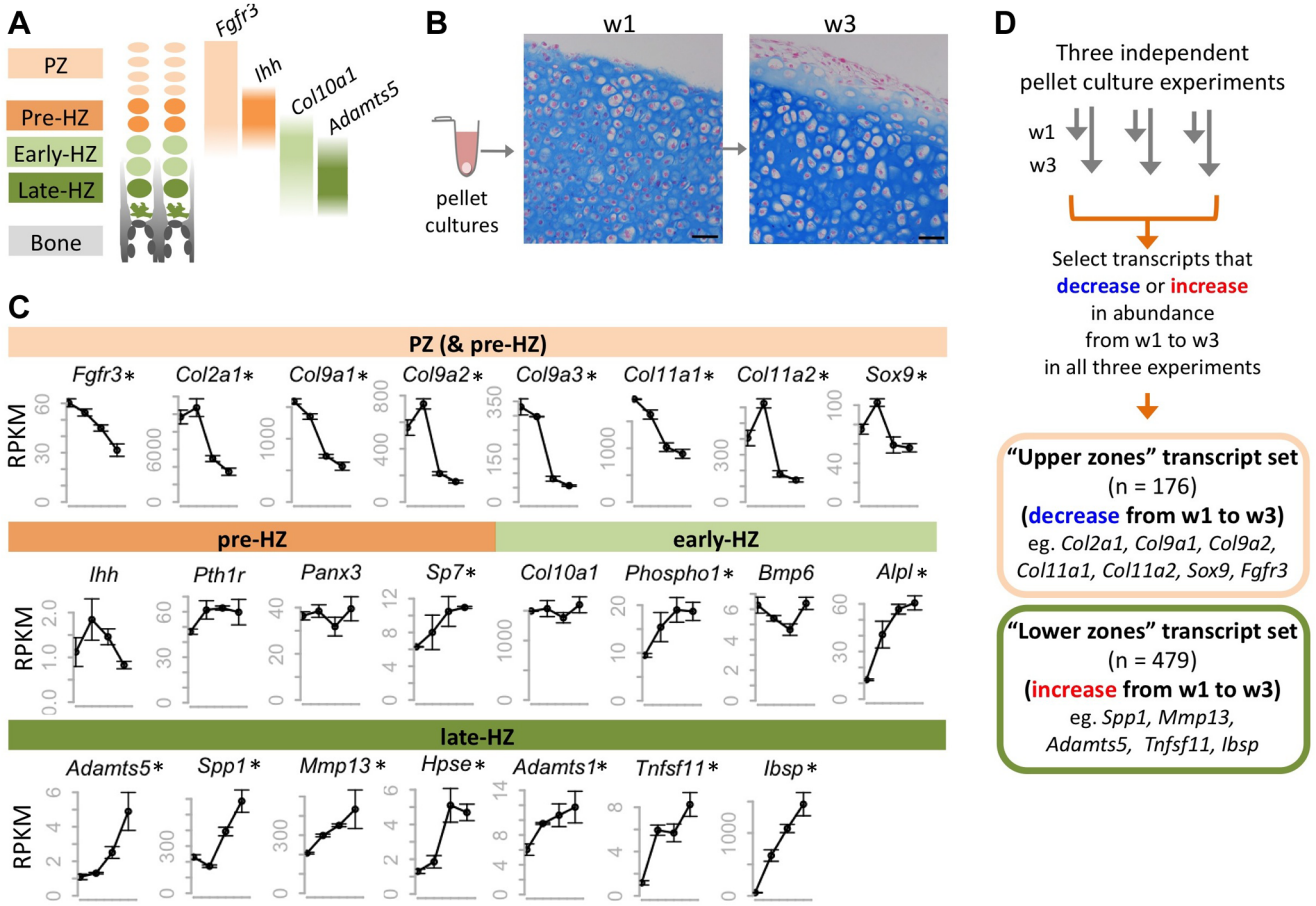
Identifying transcripts associated with the maturation of wild-type chondrocytes in pellet cultures. A: Schematic representation of the zones of maturation in the growth plate. Examples of four transcripts that are associated with specific maturation states are indicated. PZ: proliferative zone. HZ: hypertrophic zone. B: Photomicrographs of Alcian Blue and Nuclear Fast Red stained sections through chondrocyte pellets after 1 week (w1) and 3 weeks (w3) in culture (scale bar  =  50 µm). Note the overall increase in chondrocyte size between w1 and w3, consistent with chondrocytes undergoing hypertrophy. C: Graphs showing the abundance (RPKM) of selected transcripts in wild-type chondrocytes after 4 days, or 1, 2 or 3 weeks in pellet culture. Above each graph, the growth plate zones in which transcripts have previously been shown to be most abundant are indicated. RNA-seq was performed on 3 pellets per time point, and the average RPKM at each time point is indicated (± 1 SD). Transcripts that significantly (p<0.05) increase or decrease in abundance over time are indicated with an asterisk. D: Flow Chart showing the method used to define a set of transcripts that consistently decreased or increased in abundance over time in three independent experiments.

To establish a system in which the molecular consequences of SHP2 depletion in chondrocytes could be studied, we utilized mouse primary chondrocyte pellet cultures [16],[17]. In the growth plate, chondrocytes proceed through distinct zones of maturation before their cartilage is degraded and replaced by bone (Figure 1A). The proliferative zone (PZ) contains small, rapidly dividing chondrocytes that form columns, while the hypertrophic zone (HZ) contains chondrocytes that have exited the cell cycle and dramatically increased in size (Figure 1A) [18]. In chondrocyte pellet cultures, the transition from the proliferative to hypertrophic state can be modeled (Figure 1B). We evaluated the process of chondrocyte maturation at the transcriptional level by performing RNA-seq on pellet cultures.

To verify that RNA-seq is a reproducible method for measuring transcript abundance in chondrocytes undergoing maturation in pellet cultures, we first performed RNA-seq on wild-type chondrocytes after 4 days, or 1, 2 or 3 weeks in culture. We sequenced 3 libraries per time point, and obtained, on average, 13 million 50-bp paired-end reads per library, of which >95% could be mapped to the mouse genome (Table S1). To determine the relative abundance of transcripts at each time point, we counted the number of reads mapping to each transcript, and normalized this based on the length of the transcript and the number of reads per library, to obtain an RPKM value [19] (Dataset S1). We found that the abundance of transcripts was similar across libraries from the same time-point (R^2^>0.99) (Figure S1), and that the most abundant transcripts corresponded to genes known to be highly expressed in chondrocytes (e.g., *Col2a1*, *Col9a1*, *Sparc*) [20]. Furthermore, while transcripts associated with intermediate stages of chondrocyte maturation (pre-hypertrophic and early-hypertrophic) did not consistently change in abundance over time, many transcripts known to be associated with proliferative chondrocytes decreased in abundance and transcripts associated with late-hypertrophic chondrocytes increased in abundance (Figure 1C; Table S2). This indicates that as pellet cultures mature, the proportion of proliferative chondrocytes decreases, while the proportion of terminally-differentiated chondrocytes increases. Thus, our data suggest that RNA-seq is a reliable method for detecting changes in transcript abundance that occur as chondrocytes mature from the proliferative through to the late-hypertrophic stage.

To further define transcriptional changes associated with chondrocyte maturation, we sought to identify a reproducible set of transcripts that change in abundance over time. First, we selected all transcripts that significantly increased or decreased in abundance over time in our wild-type pellet cultures. Only transcripts with an RPKM >3 that showed at least a 1.6-fold change and a *p* value <0.05 were selected. Second, to reduce false positives, we incorporated data from 2 additional independent RNA-seq data sets. These were from pellets that were used as vehicle controls in our experiments described in further detail below. We justified incorporating data from vehicle-treated pellets by showing that vehicle-treatment alone did not affect known chondrocyte maturation transcripts (Figure S2) and that the correlation coefficient between untreated and vehicle-treated libraries at the same time point was high (R^2^ > 0.98) (Figure S1). We found that 81% of all transcripts that increased or decreased in abundance over time in untreated pellets also increased or decreased in abundance by at least 1.3 fold in both sets of vehicle-treated pellets. By focusing only on the transcripts that reached statistical significance and exhibited at least a 1.6-fold change in all three experiments, we identified a high-confidence set of 176 transcripts that consistently decreased in abundance over time (“upper zones” transcript set) and a set of 479 transcripts that consistently increased in abundance (“lower zones” transcript set) (Figure 1D, Dataset S1). Known markers of proliferative or hypertrophic chondrocytes were identified in these transcript sets, respectively, thus validating our approach (Figure 1D). Conversely, many of the transcripts in these data sets have not yet been studied in the growth plate and likely represent novel factors involved in chondrocyte maturation.

### Shp2 Depletion Increases The Abundance Of Transcripts Associated With Early Maturation Stages, And Decreases The Abundance Of Transcripts Associated With Terminal Differentiation

**Figure 2:**
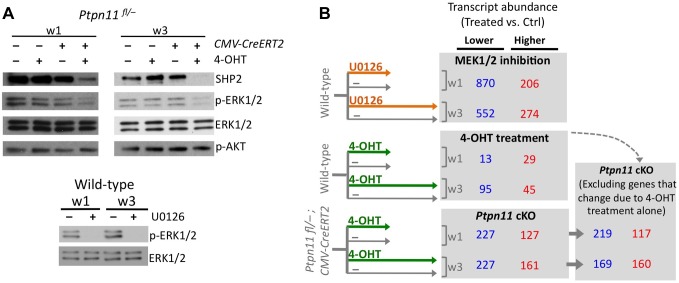
Identifying transcripts that change in abundance following SHP2 depletion or MEK1/2 inhibition in pellet cultures. A: Immunodetection of SHP2, phospho-ERK1/2, total ERK1/2, and phospho-AKT in chondrocyte pellet cultures. Top: pellets from *Ptpn11^fl/–^* or *Tg*(*CMV-CreERT2); Ptpn11^fl/–^* mice were treated daily with 4-OHT or vehicle control (EtOH, -) and harvested after 1 and 3 weeks. Bottom: pellets from wild-type mice were treated daily with a MEK1/2 inhibitor (U0126), or vehicle control (DMSO, -), and harvested after 1 and 3 weeks. B: Number of transcripts that had significantly lower (blue) or higher (red) abundance in treated pellets compared to vehicle treated pellets, either at w1 or w3. Transcripts that changed in abundance in *Ptpn11* cKO pellets were excluded if they exhibited similar changes in wild-type pellets treated with 4-OHT.

We performed RNA-seq on pellets carrying a conditional *Ptpn11* allele and a tamoxifen-dependent Cre recombinase, that were treated daily with 4-hydroxytamoxifen (4-OHT), to inactivate *Ptpn11* (*Ptpn11* cKO), or with vehicle control (0.1% v/v EtOH). One week of 4-OHT treatment (w1) induced recombination in >90% of chondrocytes (Figure S3) and reduced SHP2 levels (Figure 2A). Two weeks of 4-OHT treatment, followed by an additional week in culture (w3), almost completely depleted SHP2 (Figure 2A). Since it was not yet known whether long-term 4-OHT treatment itself would affect gene expression, we also performed RNA-seq on similarly treated wild-type pellets (Figure 2B). Of all transcripts that changed in abundance in *Ptpn11* cKO pellets, 18 (5%) at w1 and 59 (15%) at w3 also changed in wild-type pellets treated with 4-OHT. We therefore excluded these 77 transcripts from subsequent analyses (Figure 2B, Figure S4). There was significant (p<0.001) overlap between transcripts whose abundance changed in *Ptpn11* cKO pellets at w1 and w3 (Figure S5). However, there were also differences between the w1 and w3 data sets. For example, transcripts encoding protein translation components were decreased in w1 but not w3 in *Ptpn11* cKO pellets (Figure S6). Thus, during chondrocyte maturation the transcriptional response to short term versus sustained SHP2 depletion is similar but not identical.

**Figure 3:**
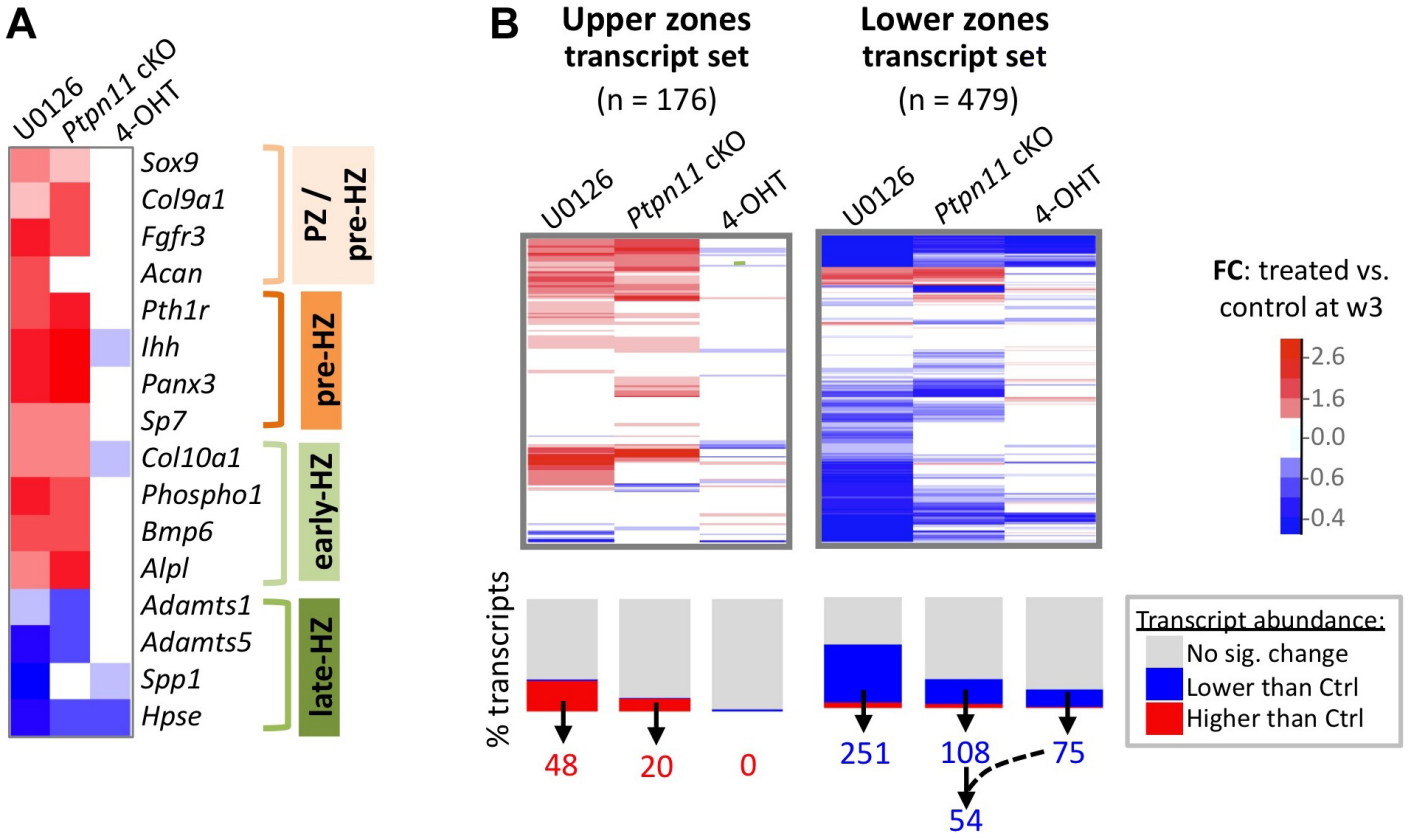
SHP2 depletion or MEK1/2 inhibition increases the abundance of PZ, pre-HZ and early-HZ transcripts, and decreases the abundance of late-HZ transcripts. A: Heat map showing the fold change in abundance (treated vs. control pellets at w3) of selected transcripts that are associated with a specific stage of chondrocyte maturation. B: Heat maps showing the fold change in abundance (treated vs. control pellets at w3) for all transcripts that we included in the “upper zones” and “lower zones” transcript sets. The graph below the heat map indicates the percentage of transcripts for which the increase (red) or decrease (blue) in abundance was significant (p<0.05). The number of “upper zones” transcripts that increased in abundance in treated pellets (red), and the number of “lower zones” transcripts that decreased in abundance in treated pellets (blue), is indicated below. For *Ptpn11* cKO pellets, of the 108 “lower zones” transcripts that significantly decreased in abundance, 54 did not significantly change in wild-type pellets treated with 4-OHT.

To assess the effect of SHP2 depletion on chondrocyte maturation, we compared transcript abundance in *Ptpn11* cKO pellets to that of vehicle pellets at w3. Transcripts expressed by proliferative through early-hypertrophic chondrocytes were increased and transcripts expressed by late stage hypertrophic chondrocytes were decreased in *Ptpn11* cKO pellets (Table S3; Figure 3A). The greatest change in transcript abundance following SHP2 depletion was in the pre-hypertrophic chondrocyte expressed transcript *Ihh* (6-fold increase). However, not all well-characterized stage-specific transcripts were altered by SHP2 depletion. Therefore, we took a non-biased approach by examining the effect of SHP2 depletion on the “upper zones” and “lower zones” transcript sets we had previously defined in wild-type pellet cultures (Figure 1D). Compared to control pellets at w3, *Ptpn11* cKO pellets had 44% of the “upper zones” transcripts increase in abundance by > 1.3 fold, and 34% of the “lower zones” transcripts decrease in abundance by > 1.3 fold. When considering *Ptpn11* cKO transcripts whose change in abundance satisfied our significance criteria (1.6-fold change, p<0.05) 20 “upper zones” transcripts were increased and 54 “lower zones” transcripts were decreased (Figure 3B; Dataset S1; Figure S7). These data are consistent with SHP2 depletion delaying terminal differentiation while maintaining chondrocytes in the proliferative, pre-hypertrophic, or early-hypertrophic stage.

### Mek1/2 Inhibition Perturbs Chondrocyte Maturation In A Similar Manner To Shp2 Depletion

SHP2 has been proposed to positively regulate the ERK1/2 pathway in chondrocytes [11],[12]. Indeed, we observed reduced phospho-ERK1/2 levels after SHP2 depletion in pellet cultures (Figure 2A). To determine whether this reduction in ERK1/2 pathway activity mediates the transcriptional changes that occur following SHP2 depletion, we performed RNA-seq on pellets cultured for 1 or 3 weeks in the presence of either a vehicle control (0.1% v/v DMSO) or a MEK1/2 inhibitor (U0126) (Figure 2A, B). When considering the transcripts that changed in abundance after SHP2 depletion (excluding transcripts that changed after 4-OHT treatment alone), we found that 177 (53%) and 116 (35%) had similar changes after U0126-treatment at w1 and w3, respectively (Figure 2B, Figure S8). The observed overlap is significantly greater than expected by chance alone (p<0.001). As with SHP2 depletion, we found that U0126-treatment increased the abundance of transcripts associated with the PZ, pre-HZ and early-HZ, with the most substantial changes involving the pre-HZ transcripts, *Ihh* and *Panx3* (> 3-fold increase) (Table S3; Figure 3A,B). Furthermore, we found that U0126-treatment decreased the abundance of late-HZ transcripts associated with matrix degradation, apoptosis, angiogenesis and osteoclastogenesis (Figure 3A,B; Figure S7). In particular, a gene ontology analysis revealed that transcripts with reduced abundance in U0126-treated pellets were significantly enriched for proteins involved in matrix degradation and remodeling (Figure S6). We used quantitative reverse transcription PCR (qRT-PCR) to confirm that 3 weeks of U0126-treatment increased the abundance of pre-HZ and early-HZ markers (*Ihh*, *Phospho1*) and decreased the abundance of a late-HZ marker (*Spp1*) (Figure S9). Altogether, these data indicate there is substantial overlap between the genes regulated by SHP2 and MEK1/2 in chondrocytes, and that MEK1/2 inhibition and SHP2 depletion have similar effects on chondrocyte maturation.

### RNA-Seq Identifies Transcription Factors With Known Roles In Chondrocyte Maturation, And Suggests Novel Transcription Factors That May Be Involved In This Process

**Figure 4:**
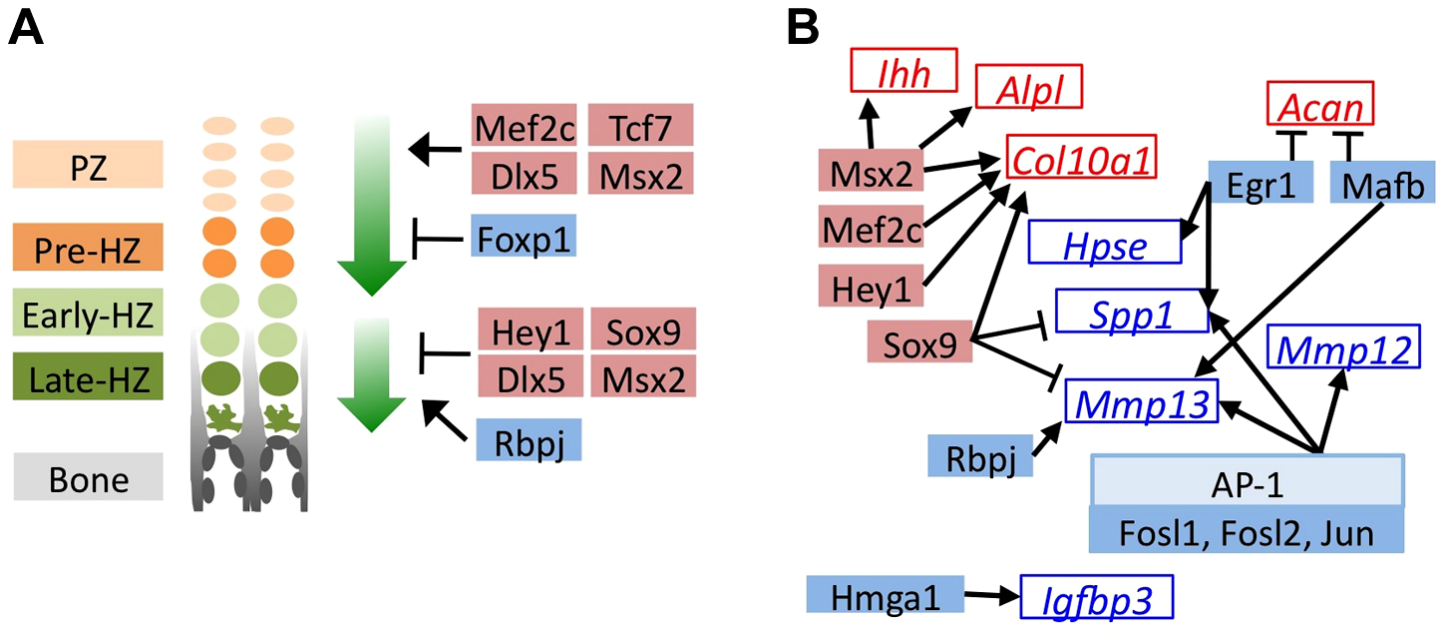
MEK1/2 inhibition or SHP2 depletion alters the expression of transcription factors with known roles in chondrocyte maturation. Schematic diagrams of genes encoding transcription factors (solid boxes) or their known target genes (empty boxes) that significantly decreased in abundance (blue) or increased in abundance (red) in U0126-treated pellets at w3. All transcription factors also showed similar increases or decreases in *Ptpn11* cKO pellets at w3, but not all reached statistical significance. A: Transcription factors with known roles in promoting chondrocyte hypertrophy (upper green arrow), or in promoting the terminal differentiation of chondrocytes and/or their replacement by bone (lower green arrow). B: Transcription factors whose known target genes also significantly changed in abundance in U0126-treated pellets. Arrows indicate transcriptional activation while bar-headed lines indicate transcriptional repression.

To identify the transcription factors that may mediate the perturbation of chondrocyte maturation observed in U0126-treated and SHP2-depleted pellets, we selected the 57 transcription factors that had increased or decreased abundance in U0126-treated or SHP2-depleted pellets at w3, and were unaffected in wild-type pellets treated with 4-OHT (Figure S10). We identified several transcription factors that may be responsible for maintaining the expression of genes associated with the pre-hypertrophic and early-hypertrophic stages, and for repressing the expression of genes associated with the late-hypertrophic stage, in U1026-treated or SHP2-depleted pellets. In particular, we observed increased expression of transcription factors known to promote hypertrophy and/or inhibit terminal differentiation (*Mef2c, Tcf7, Dlx5, Msx2, Hey1, Sox9*), and decreased expression of transcription factors known to inhibit hypertrophy and/or promote terminal differentiation (*Foxp1, Rbpj*) (Figure 4A, Table S4). In addition, we identified several transcription factors whose known target genes were also affected by MEK1/2 inhibition or SHP2 depletion, such as *Egr1* and its target genes *Spp1* and *Hpse*, which all decreased in abundance following MEK1/2 inhibition (Figure 4B, Table S5). We selected 3 transcription factors for qRT-PCR validation (*Hmga2, Fols1, Egr1*) and verified that they were less abundant following U0126-treatment (Figure S9). Thus, we have identified multiple transcription factors with known roles in the growth plate that may act downstream of SHP2 and ERK1/2 during the maturation and terminal differentiation of chondrocytes. This suggests that other transcription factors identified by RNA-seq (Figure S10) also have roles in chondrocyte maturation or terminal differentiation.

### Mosaic Inactivation Of *Ptpn11 In Vivo* Alters Chondrocyte Maturation, Disrupts The Normal Organization Of Growth Plates, And Leads To Enchondroma-Like Lesions That Contain Mixtures Of *Ptpn11* Mutant And Non-Mutant Chondrocytes

**Figure 5:**
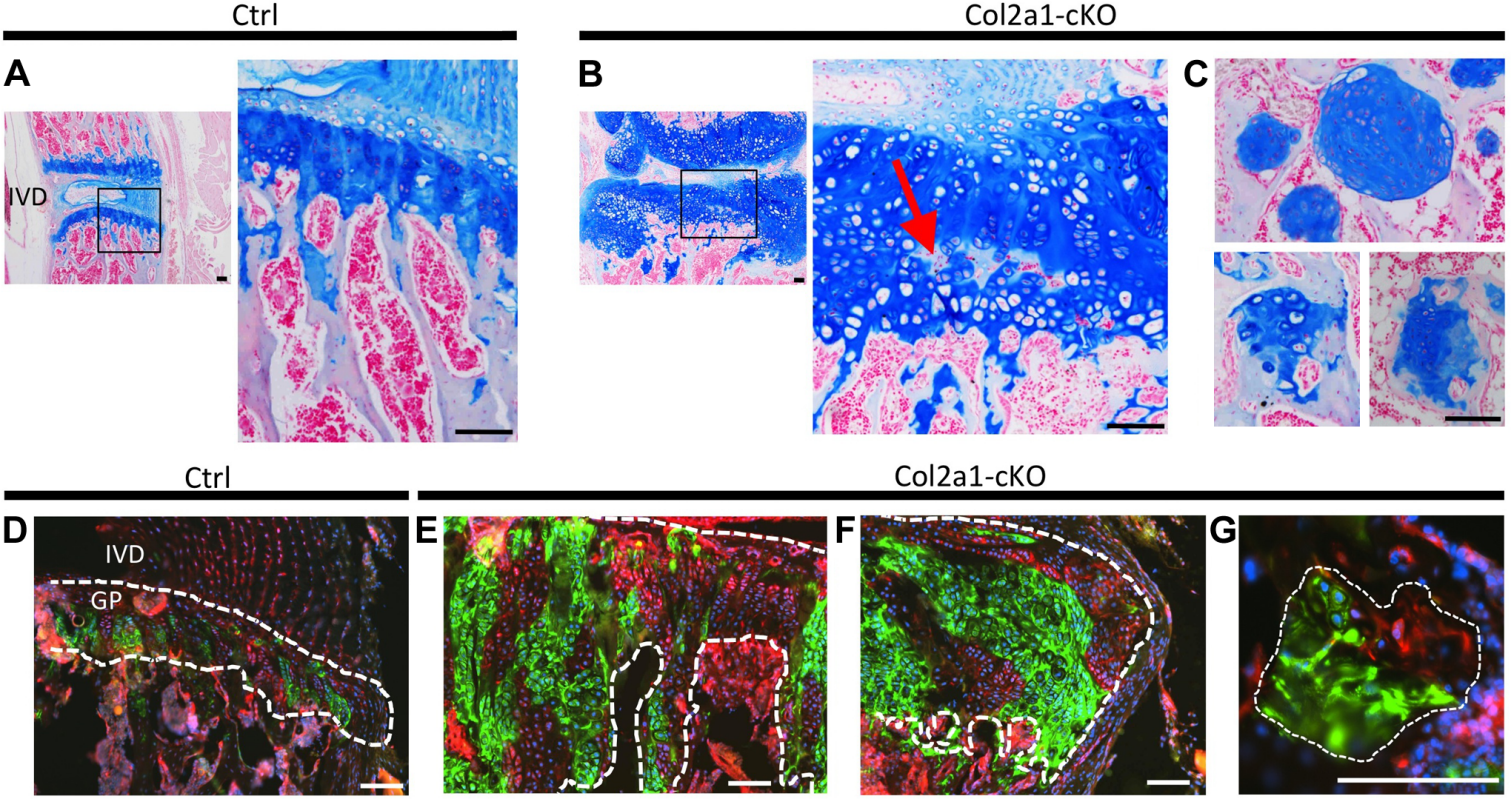
Lineage tracing of SHP2-depleted chondrocytes in mouse vertebral growth plates following mosaic postnatal *Ptpn11* inactivation. Tissue sections of lumbar vertebral growth plates (GP) and intervertebral disks (IVD) from *Tg*(*Col2a1-CreER); Rosa26^mTmG/+^*;*Ptpn11^fl^* ^/*fl*^ (Col2a1-cKO) or *Tg*(*Col2a1-CreER); Rosa26^mTmG/+^*;*Ptpn11^fl/+^* (Ctrl) mice that had been administered tamoxifen for 5 days, starting at w1, and sacrificed at w7. A-C: Tissue sections stained with Alcian Blue and Nuclear Fast Red. In Col2a1-cKO mice, the vertebral growth plates are enlarged and disorganized, with ectopic areas of ossification inside the growth plate (red arrow in B) and enchondroma-like lesions below the growth plate (C). D-G: Merged fluorescent images showing cells in which the *mTmG* reporter allele has (green fluorescence) or has not (red fluorescence) been recombined by Cre recombinase. Note that approximately 50% of growth plate chondrocytes fluoresce green, and in Col2a1-cKO mice, the expanded regions of the growth plate (E), lateral outgrowths (F) and enchondroma-like lesions (G) contain both green and red fluorescing cells. Dashed-white lines outline the cartilage. Scale bars  =  100 µm.

Since SHP2 depletion perturbed chondrocyte maturation in pellet cultures, we inactivated *Ptpn11 in vivo* and determined the effect on chondrocyte maturation. It has recently been reported that postnatal inactivation of *Ptpn11* in chondrocytes alters the thickness and organization of vertebral growth plates [13], but it was not shown on a molecular level whether the process of chondrocyte maturation was altered. We used a tamoxifen-inducible chondrocyte *Cre* driver (*Col2a1-CreER*) to inactivate *Ptpn11* postnatally. Mice were treated with tamoxifen for 5 days, starting at week 1, and analyzed at weeks 3, 7 and 10. A clear expansion of the vertebral growth plates, osteophyte-like lateral cartilaginous outgrowths, enchondroma-like cartilage islands below the growth plate, and an increase in chondrocyte cell size were observed by weeks 7 and 10 (Figure 5A-C). The severity of the phenotype was variable between mice (Figure S11). The enchondroma-like lesions likely arose from chondrocytes displaced from the growth plate due to ectopic ossification in upper regions of the growth plate (Figure 5B). By week 10, some growth plates had been completely resorbed while other growth plates remained expanded (Figure S11). Analysis of the *mTmG* reporter allele indicated that 5 days of tamoxifen treatment had induced recombination in ∼50% of chondrocytes (Figure 5D). Importantly, not all chondrocytes in the cartilage outgrowths and enchondroma-like lesions had evidence of Cre-mediated recombination, suggesting that the lesions contained mixtures of *Ptpn11* mutant and non-mutant chondrocytes (Figure 5E-G). Thus, our histological and lineage tracing results indicate that mosaic inactivation of *Ptpn11* in growth plates affects mutant and wild-type chondrocytes; the latter most likely due to altered paracrine signaling.

**Figure 6:**
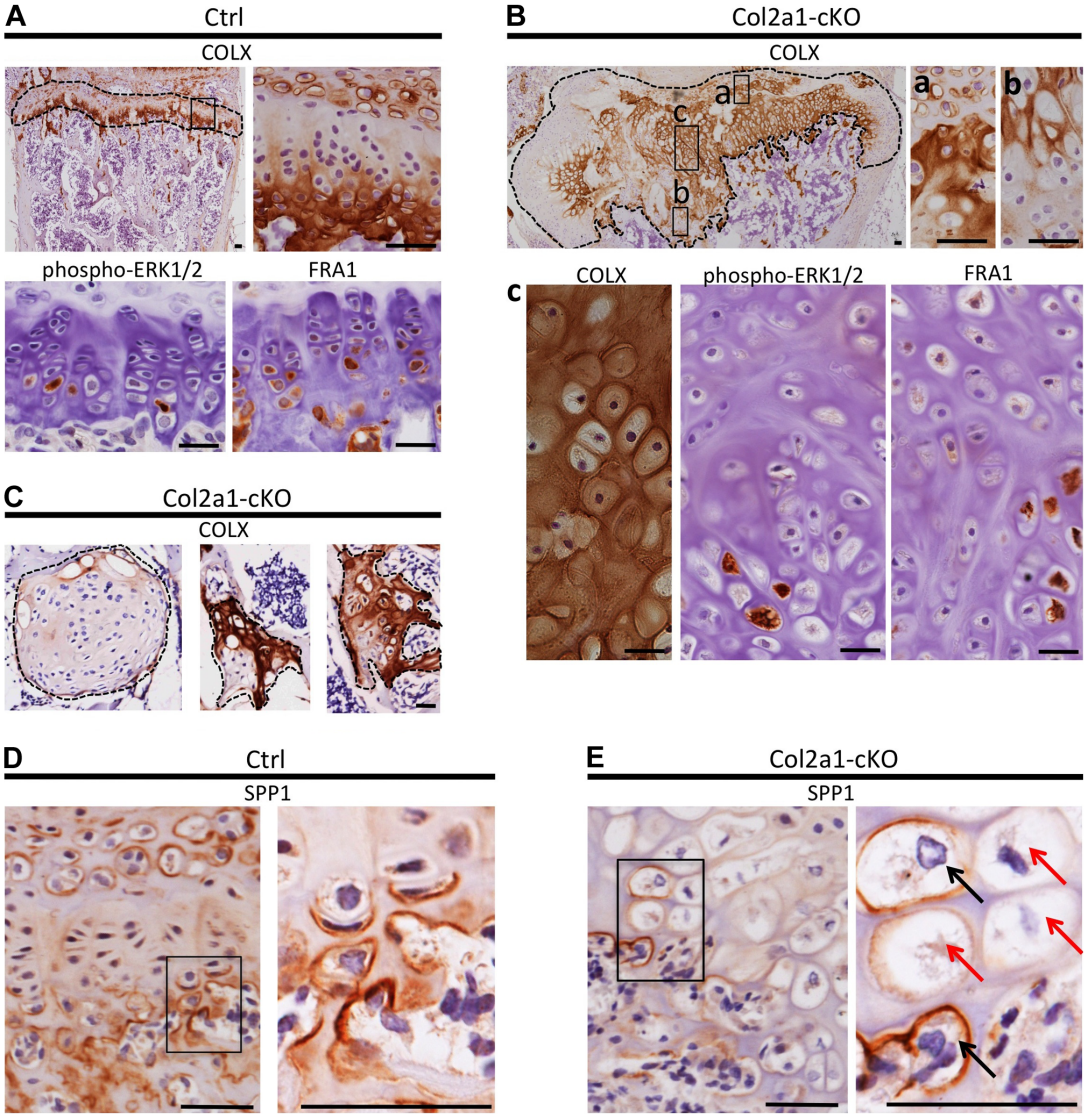
Disordered maturation zones and delayed terminal differentiation in mouse vertebral growth plates following mosaic postnatal inactivation of *Ptpn11* in chondrocytes. Immunohistochemistry performed on tissue sections from the vertebral growth plates of Ctrl mice (A,D) or the growth plates (B,E) or enchondroma-like lesions (C) in Col2a1-cKO mice. A: Ctrl growth plate showing COLX, p-ERK1/2 and FRA1 immunoreactivity in the hypertrophic zone. COLX immunoreactivity is also observed in the calcifying region of the annulus fibrosus that lies directly above the growth plate. B: Col2a1-cKO growth plate showing ectopic COLX-positive clusters at the top of the growth plate (a) and ectopic COLX-negative clusters at the bottom of the growth plate (b). Furthermore, hypertrophic chondrocytes within the center of the growth plate display disorganized, scattered p-ERK1/2 and FRA1 immunoreactivity (c). C: Enchondroma-like lesions contain both COLX-positive and COLX-negative chondrocytes. D: Ctrl growth plate showing SPP1 immunoreactivity at the bottom of the hypertrophic zone and in mineralizing annulus fibrosus cells. E: In Col2a1-cKO growth plates, expanded regions of SPP1-negative hypertrophic chondrocytes are observed (red arrows) and only a few scattered hypertrophic chondrocytes are SPP1-positive (black arrows). IHC tissue sections were counterstained with hematoxylin. Scale bars  =  25 µm.

To determine whether mosaic *Ptpn11* inactivation alters the normal organization of chondrocytes into distinct maturation zones we performed immunohistochemistry (IHC) for type 10 collagen (COLX), which is produced by early-hypertrophic chondrocytes [18]. As expected, in control mice, COLX was localized to the matrix surrounding only the lowest ∼3 cells in each chondrocyte column (Figure 6A). In contrast, the distribution of COLX was altered in *Ptpn11* cKO mice. Their growth plates had regions in which the height of the COLX-positive zone was expanded to >10 cells (Figure 6B). Furthermore, the organization of the COLX-positive cells was altered, with ectopic COLX-positive clusters in upper growth plate zones, ectopic COLX-negative clusters in lower growth plate zones, and COLX-positive chondrocytes in the lateral outgrowths that were arranged into columns oriented perpendicular to the main axis of the growth plate (Figure 6B). Both COLX-positive and COLX-negative chondrocytes were detected in the enchondroma-like lesions (Figure 6C). These data indicate that the organization of chondrocytes into distinct growth plate zones is disrupted, and that the lateral outgrowths and enchondroma-like lesions contain both mature and immature chondrocytes.

To further characterize the process of chondrocyte maturation, we examined the distribution of phospho-ERK1/2, which is normally detected in the hypertrophic zone [21], and FRA1, a transcription factor encoded by *Fosl1*, which decreased in abundance following SHP2 depletion in pellet cultures (Figure 4B). FRA1 and p-ERK1/2 were detected in hypertrophic chondrocytes in control mice (Figure 6A), but were distributed in a scattered, disorganized fashion in the hypertrophic zone of *Ptpn11* cKO mice (Figure 6B). In addition, we observed minimal staining for the terminal differentiation marker osteopontin (SPP1) (Figure 6D,E) [18], suggesting that the hypertrophic zone was expanded due to an increase in the number of chondrocytes that had not yet reached the late-hypertrophic stage. We did not detect significant differences in osteoclast recruitment or vascular invasion (Figure S12). In summary, the normal distribution pattern of stage-specific matrix components and signaling pathway components is altered in *Ptpn11* cKO growth plates, and the observed delay in the terminal differentiation of hypertrophic chondrocytes is consistent with our pellet culture data. Together, our data suggest that enchondromas arise from clusters of chondrocytes that are displaced from the growth plate due to delayed terminal differentiation and disrupted growth plate architecture.

### Disorganized Chondrocyte Maturation Zones Are Also Observed In Excised Human Mc Exostoses

**Figure 7:**
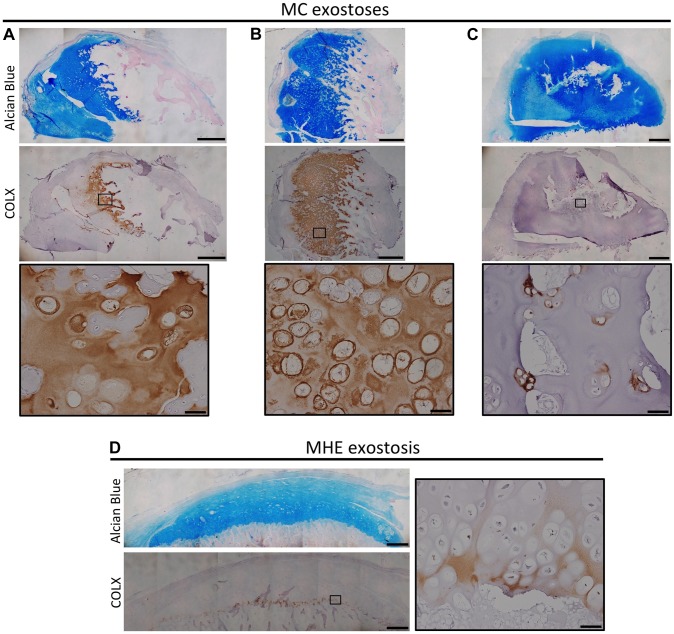
Disorganized distribution of COLX in exostoses from MC patients. Alcian Blue and Nuclear Fast Red staining (upper) and immunohistochemical staining of COLX (lower) in exostoses from MC patients (A-C) or an MHE patient (D). Note that the MHE exostosis shows a thin layer of COLX staining at the base of the cartilage cap (D). In contrast, in MC exostoses, the distribution of COLX staining is variable and disorganized, with some exostoses showing expanded COLX domains (A,B), and other showing scatted COLX-positive chondrocytes in the center of the lesion (C). IHC tissue sections were counterstained with hematoxylin. Scale bar  =  1mm; Scale bar in insets  =  50 µm.

We next determined whether chondrocyte maturation and organization is altered in cartilage lesions from MC patients who carry mutations in *PTPN11*. We observed a variable and disorganized distribution of COLX in MC exostoses. In particular, some lesions had intense and expanded regions of COLX immunoreactivity (Figure 7A-B), while other regions had isolated disorganized clusters of COLX immunoreactivity (Figure 7C). We compared this to MHE exostoses, which have cartilage caps that display relatively normal growth plate architecture [1]. We found that MHE exostoses have a narrow zone of COLX immunoreactivity at the base of the cartilage cap, as previously reported [22],[23] (Figure 7D). Thus, in contrast to MHE exostoses, lesions from MC patients display disorganized chondrocyte maturation zones.

### Inactivation Of *Ptpn11* In Fibroblasts Induces Exostosis-Like Outgrowths And Ectopic Cartilage On Bone Surfaces

**Figure 8:**
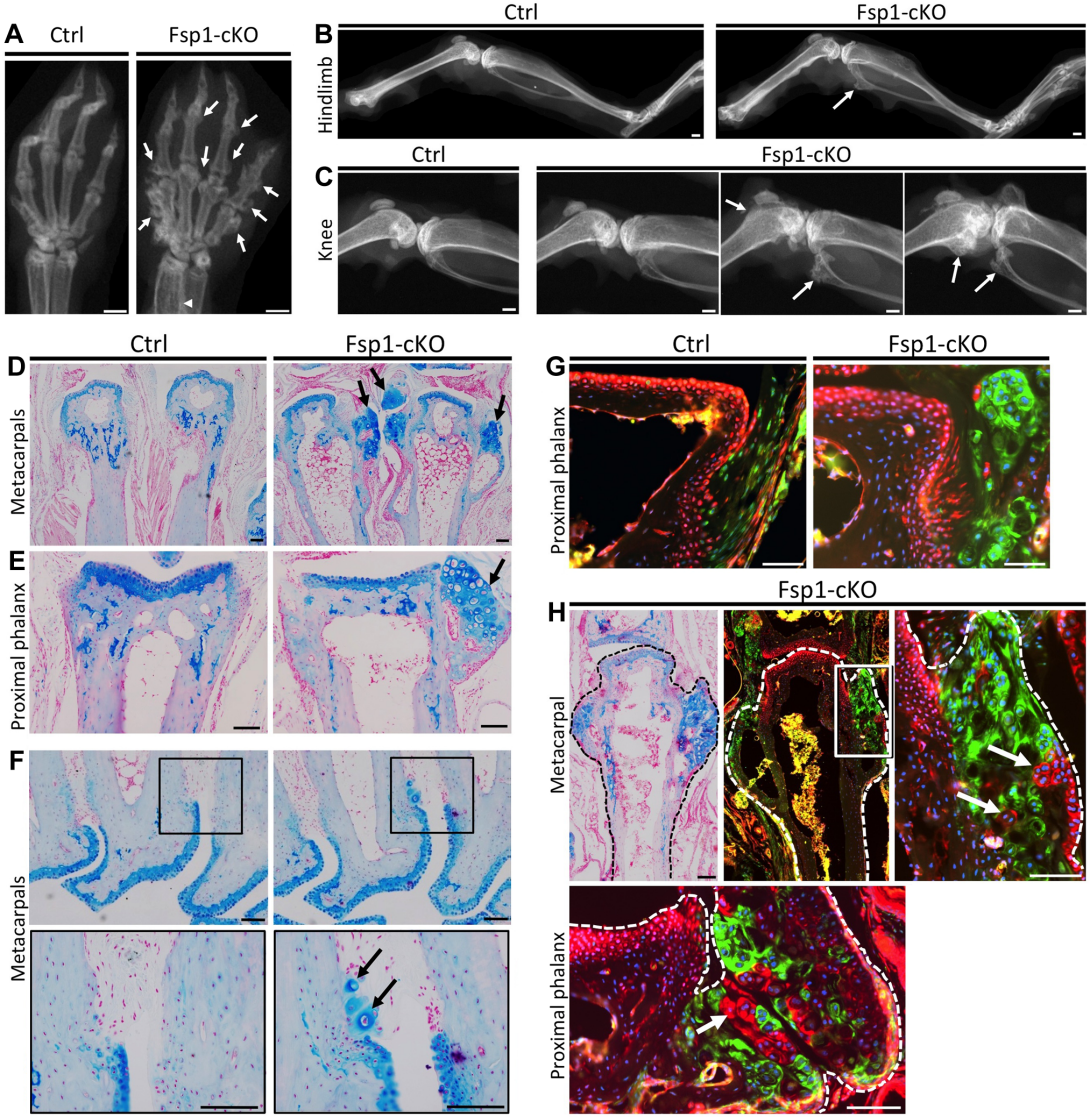
Inactivation of *Ptpn11* in *Fsp1-Cre*-expressing fibroblasts induces ectopic cartilage formation. A-C: Radiographs of the hands (A), hindlimbs (B) and knees (C) of 20 week old Ctrl (*Fsp1-Cre; Ptpn11^fl/+^; Rosa26^mTmG/+^*) and Fsp1-cKO (*Fsp1-Cre; Ptpn11^fl/fl^; Rosa26^mTmG/+^*) mice. All Fsp1-cKO mice (n = 6) developed multiple exostosis-like outgrowths on the metacarpals and phalanges (arrows, A). Incompletely penetrant exostosis-like outgrowths were observed on the fibula, tibia and femur (arrows, B-C). D-F: Alcian Blue and Nuclear Fast Red stained sections of the metacarpals (D, F) and proximal phalanges (E) in Ctrl and Fsp1-cKO mice at w20. Arrows indicate exostosis-like outgrowths (D, E) and ectopic clusters of chondrocytes on the bone surface (F). G-H: Merged fluorescent images of the proximal phalanges of Ctrl and Fsp1-cKO mice (G) and a metacarpal and proximal phalynx of an Fsp1-cKO mouse (H). In Ctrl mice, cells with a history of *Fsp1-Cre* expression (green) are observed at the bone-ligament attachment site, while evidence of Cre-mediated recombination is not observed in chondrocytes or osteoblasts (red). In Fsp1-cKO mice, cells with a history of *Fsp1-Cre* expression (green) have acquired the round morphology of chondrocytes. Note that that not all chondrocytes within the ectopic cartilaginous outgrowths have evidence of Cre-mediated recombination (red cells, arrows in H). Scale bars in A-C  =  1 mm; D-H  =  100 µm.

Inactivation of *Ptpn11* in perichondrial progenitor cells within the Groove of Ranvier induces ectopic chondrogenesis and leads to the formation of exostosis-like outgrowths [11]. To determine whether SHP2 also regulates the cell fate of other mesenchymal cell populations, we utilized *Fsp1-Cre* [24]–[27] to inactivate *Ptpn11* in fibroblasts (Fsp1-cKO). At 3 weeks of age, Fsp1-cKO mice had no overt skeletal defects (Figure S13). At 20 weeks of age, exostosis-like outgrowths were observed on the metacarpals and phalanges of the hands of all Fsp1-cKO mice examined (n = 6) (Figure 8A), and the fibula, tibia, femur and/or calcaneus of some Fsp1-cKO mice (Figure 8B,C). Histological analysis revealed that ectopic cartilage was found at bone-ligament attachment sites at the ends of the metacarpals and phalanges (Figure 8D-H), on the cortical surfaces of femoral and tibial bone (Figure S14A), in the interosseous membrane between the radius and ulna (Figure S13B), and adjacent to ligament-attachment sites on the articular surface of the tibia (figure S14B,C). We found that *Fsp1-Cre* drives recombination in fibroblasts at bone-ligament attachment sites (Figure 8G, Figure S14C). Lineage tracing in Fsp1-cKO mice indicated that the ectopic cartilaginous growths contained mixtures of recombined and un-recombined chondrocytes (Figure 8H), raising the possibility that SHP2-deficient cells can signal to neighboring wild-type cells and induce them to inappropriately undergo chondrogenesis. In summary, we have identified a novel cell population that can undergo chondrogenesis following loss of SHP2.

## Discussion

**Figure 9:**
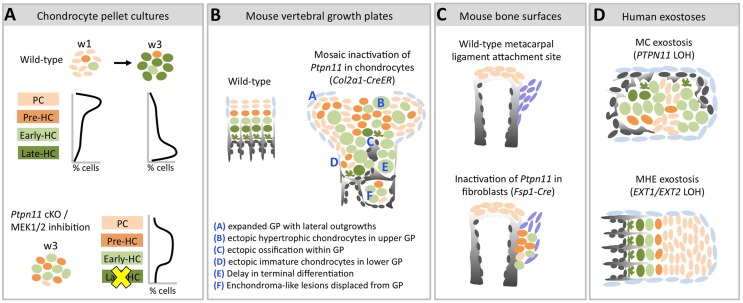
Model of the consequences of SHP2 depletion in pellet cultures, mouse vertebral growth plates, periarticular fibroblasts and human MC exostoses. Schematic diagrams showing chondrocytes (color-coded) at different stages of maturation (PC  =  proliferative chondrocyte. HC  =  hypertrophic chondrocyte). A: In wild-type pellets, most chondrocytes are in the proliferative stage at w1, and transition to the late-hypertrophic stage by w3. *Ptpn11* cKO or U0126-treated pellets have more pre-HC and early-HC transcripts and fewer late-HC transcripts compared to control pellets at w3. This suggests that SHP2 depletion or MEK1/2 inhibition delays the entry of chondrocytes into the late-hypertrophic stage, thus leading to an accumulation of chondrocytes in the pre-hypertrophic and early-hypertrophic stages. B: Mosaic postnatal inactivation of *Ptpn11* in vertebral growth plates disrupts the normal organization of chondrocytes into distinct zones, leads to lateral outgrowths and enchondroma-like lesions. The scarcity of chondrocytes in the late-hypertrophic stage and the abundance of chondrocytes in the early-hypertrophic stage suggest that loss of SHP2 delays the transition from the early to late-hypertrophic stage, thereby delaying terminal differentiation. C: Inactivation of *Ptpn11* in fibroblasts leads to ectopic cartilage formation at bone-ligament attachment sites. D: Hypertrophic chondrocytes are variably distributed in MC exostoses. This is in contrast to MHE exostoses, which have a cartilage cap that resembles a normal growth plate.

Here, we investigate the mechanisms by which loss of SHP2 drives enchondroma and exostosis formation. Firstly, by analyzing the transcriptome of chondrocytes undergoing maturation *in vitro*, we show that loss of SHP2 or inhibition of the ERK1/2 pathway delays chondrocyte terminal differentiation (Figure 9A). Secondly, we show that enchondromas in mosaic SHP2-deficient mice arise from chondrocytes displaced from the growth plate due to delayed terminal differentiation and disrupted growth plate architecture (Figure 9B). Thirdly, we create a novel mouse model of exostosis formation, by inactivating *Ptpn11* in *Fsp1-Cre*-expressing fibroblasts (Figure 9C). Finally, we show that mouse enchondromas and exostoses can contain mixtures of wild-type and SHP2-deficient chondrocytes. Together, our results suggest that reduced ERK1/2 signaling, altered cell fate decisions, impaired chondrocyte terminal differentiation, disorganized growth, and altered paracrine signaling contribute to the pathophysiology of MC.

To investigate the mechanisms by which mosaic loss of SHP2 causes chondrocytes to be displaced from the growth plate and give rise to enchondromas, we studied the role of SHP2 in chondrocyte maturation and growth plate organization. By applying massively parallel RNA-seq technology to comprehensively evaluate the transcriptome of chondrocytes undergoing maturation in pellet cultures, we show that SHP2-depletion increases the abundance of transcripts associated with pre-hypertrophic and early-hypertrophic chondrocytes and decreases the abundance of transcripts associated with late-hypertrophic chondrocytes (Figure 3). This suggests that loss of SHP2 delays the terminal differentiation of chondrocytes from the early-hypertrophic to the late-hypertrophic stage. We provide further support for this hypothesis *in vivo* by showing that loss of SHP2 in mouse vertebral growth plates leads to an expanded domain of hypertrophic chondrocytes that have not yet terminally differentiated (Figure 6), and that lesions from human MC patients have expanded hypertrophic zones (Figure 7). In addition, analysis of mosaic SHP2-deficient growth plates indicates that loss of SHP2 disrupts the normal organization of chondrocyte maturation zones (Figure 6). As a consequence of this lack of organization, ectopic ossification occurs in upper regions of the growth plate (Figure 5), causing disorganized clusters of proliferative and hypertrophic chondrocytes to be displaced below the growth plate (Figure 6). Thus, our results suggest that both delayed chondrocyte terminal differentiation and disrupted growth plate organization contribute to the formation of enchondromas in mosaic SHP2-deficient mice.

In contrast to the expanded hypertrophic domain and disorganized maturation zones we observed in mosaic SHP2-deficient mice, other enchondromatosis mouse models (carrying *Gli2* or mutant *PTHR1* transgenes) have well organized growth plates with narrow hypertrophic zones [28],[29]. This suggests that more than one mechanism for enchondroma formation may exist. It will be of interest to determine whether mutations in *IDH1* or *IDH2*, which cause Ollier disease and Maffucci syndrome, affect chondrocyte organization and terminal differentiation in a similar manner to SHP2 depletion [7]. Furthermore, it has been proposed that enchondromas may arise due to inappropriate chondrogenesis of mesenchymal stem cells in the marrow cavity [30]; future experiments will be needed to determine whether this could be an alternate mechanism by which SHP2-deficiency induces enchondroma formation.

To investigate which cell types can give rise to exostoses, we inactivated *Ptpn11* using *Fsp1*-*Cre*, which has been shown to be expressed in fibroblasts in multiple tissue types [24]–[27]. Intriguingly, this led to ectopic cartilage formation at multiple sites along the surface of bones and at bone-ligament attachment sites (Figure 8). Prior studies have shown that loss of SHP2 in perichondrial progenitor cells induces ectopic chondrogenesis [11],[12], and that mesenchymal stem cells lacking SHP2 can undergo chondrogenesis but cannot form mature osteoblasts [31]. Together, these data suggest that SHP2 normally acts to negatively regulate the chondrogenic cell fate in multiple mesenchymal cell populations. Further research will be needed to determine whether *Fsp1-Cre* expressing cells on the bone surface are mature fibroblasts that trans-differentiate into chondrocytes following loss of SHP2, or are multipotent progenitor cells that proceed down the chondrogenic lineage following loss of SHP2.

We propose that exostoses in patients with MC develop via a fundamentally different mechanism than the exostoses in patients with MHE (Figure 9D). The growth of an MC exostosis is disordered (Figure 7). In contrast, MHE lesions have well defined cartilage caps and well organized, but aberrantly directed, growth plates [1]. Therefore, *EXT1* and *EXT2* mutations likely predispose to misdirected growth by disorienting otherwise normally functioning chondrocytes, while SHP2 mutations cause misdirected and disorganized growth by creating dysfunctional chondrocytes that signal abnormally. These differences in growth mechanism are consistent with the observation that MHE lesions do not regress but stop growing at puberty when growth plates normally close [1]. Exostoses in MC patients have been reported to regress or disappear over time [1], consistent with our observation that terminal differentiation is delayed rather than completely blocked. Therefore, when SHP2 mutant chondrocytes finally terminally differentiate they will no longer drive abnormal growth. In this regard, it will be interesting to determine whether restoration of SHP2 function will induce terminal differentiation of mutant chondrocytes and hasten regression of these lesions.

Our lineage tracing analyses suggest that SHP2-deficient and SHP2-sufficient chondrocytes can be incorporated into exostoses and enchondromas in mice (Figure 5; Figure 8). The lack of sensitive and specific anti-SHP2 antibodies for immunohistochemistry coupled with the difficulty of detecting *Ptpn11* mRNA in decalcified mouse bone precludes our conclusively showing the presence of both cell types in lesions. However, the notion that cartilage tumors can arise from a mixture of mutant and non-mutant cells is supported by the observation that enchondromas in patients with *IDH1* mutations contain both wild-type and *IDH1*-mutant chondrocytes [7], and that exostoses in *Ext1*-deficient mice contain both *Ext1*-null and wild-type chondrocytes [32]. These data suggest that altered paracrine signaling from SHP2-deficient chondrocytes can affect the specification, organization and terminal differentiation of neighboring wild-type chondrocytes.

Our data, and those of other investigators, suggest that aberrant IHH signaling contributes to cartilage tumor formation. IHH is a potent secreted regulator of chondrocyte proliferation and differentiation [33]. We observed significantly increased *Ihh* transcript abundance in SHP2-depleted and MEK1/2-inhibited pellet cultures (Figure 3). Similarly, it has previously been reported that knockdown of SHP2 in a chondrogenic cell line increases *Ihh* expression, and exostoses induced by SHP2 depletion have higher levels of *Ihh* expression than normal growth plates [11]. Also, an inhibitor of IHH signaling reduced the growth of cartilage tumors in mosaic SHP2 mutant mice [11]. However, we cannot preclude additional paracrine factors also contributing to tumor formation since transcripts for other secreted growth regulators (SOST, BMP7, BMP8A, WNT5B, WNT10B, and FGF21) exhibited increased abundance in SHP2-depleted pellet cultures. However, because increased transcript abundance does not always lead to increased protein abundance, these factors would need to be confirmed at the protein level in *vivo*. In summary, the pathogenic mechanism(s) that produce cartilage tumors in patients with MC may be complex.

In pellet cultures SHP2 depletion reduced ERK1/2 phosphorylation (Figure 2) and inhibition of ERK1/2 signaling delayed chondrocyte terminal differentiation (Figure 3). A role for ERK1/2 signaling in chondrocyte terminal differentiation is supported by prior studies showing that loss of ERK1/2 signaling *in vivo* leads to an expanded domain of hypertrophic chondrocytes [14],[15],[21]. Furthermore, loss of ERK1/2 signaling can also disrupt the organization of growth plate chondrocytes and can promote ectopic chondrogenesis in the perichondrium [14]. Together, these data suggest that the delayed chondrocyte terminal differentiation, disrupted growth plate organization, and ectopic cartilage formation in SHP2-deficient mice may all be a result of reduced ERK1/2 signaling. It would be of interest to determine whether restoring ERK1/2 signaling, such as via a constitutively active *MEK1* transgene, could prevent cartilage tumor formation in SHP2-deficient mice.

We do not know which receptor tyrosine kinases act upstream of SHP2 and the ERK1/2 pathway during chondrocyte terminal differentiation; however, EGFR1, FGFR1 and FGFR3 are likely candidates since loss of any one of these receptors in mice leads to an expansion of the hypertrophic zone [34]–[37] and loss of FGFR3 leads to enchondroma-like lesions in the long bones [36]. Furthermore, transcripts encoding several transcription factors had altered abundance after SHP2 depletion or MEK1/2 inhibition (Figure 4). Under normal circumstances, these transcription factors likely regulate terminal differentiation in response to SHP2 and ERK1/2 signaling. Of particular interest are HEY1, MSX2, DLX5 and SOX9, which have all been shown to delay the removal of hypertrophic chondrocytes *in vivo* (Figure 4) [38]–[41]. Since these four transcription factors are induced by BMP signaling [42]–[44], and the BMP pathway has been shown to inhibit terminal differentiation [45], it is possible that increased BMP signaling contributes to the delay in terminal differentiation following MEK1/2 inhibition or SHP2 depletion.

The RNA-seq datasets we identified in chondrocyte pellet cultures should be useful to other investigators interested in endochondral growth and the *ex vivo* modeling of this process. By analyzing the transcriptional changes during the maturation of wild-type chondrocytes in pellet cultures, we confirmed previously reported changes in gene expression and we identified new transcripts that likely distinguish proliferative from late-hypertrophic chondrocytes (Figure 1). However, since it is possible that some of these transcripts may be artifacts of long culture periods, future *in vivo* validation will be required to confirm their association with chondrocyte maturation. For investigators that use 4-OHT to induce Cre-mediated recombination of floxed alleles, our dataset of transcripts whose abundance changed following 4-OHT administration will also be helpful. In conclusion, by coupling *ex vivo* RNA-seq with *in vivo* conditional mouse models, we have added to our understanding of how cartilage tumors arise in patients with MC, as well as the pathways that are regulated by SHP2 during chondrocyte maturation.

## Methods

### Mouse Husbandry

The Boston Children's Hospital Institutional Animal Care and Use Committee approved these studies. The *Rosa26^mTmG^* reporter allele [46], the *Tg*(*Col2a1-CreER)* driver [47], and the Tg(*Fsp1*-*Cre*) driver [24] were purchased from The Jackson Laboratory. The *Ptpn11* floxed (*Ptpn11^fl/fl^*) and *Tg*(*CMV-CreERT2)* mice have been previously described [48],[49]. The *Ptpn11* null allele was generated by germ line deletion of the *Ptpn11* floxed allele following crossing to *actin-Cre* mice. For mosaic postnatal inactivation of *Ptpn11* in chondrocytes, 1-week-old *Tg*(*Col2a1-CreER);Rosa26^mTmG/+^;Ptpn11^fl/fl^* mice were given daily intra-peritoneal injections of 50 mg/kg tamoxifen (Sigma) suspended in corn oil for 5 days. *Tg*(*Col2a1-CreER);Rosa26^mTmG/+^;Ptpn11^fl/+^* and *Rosa26^mTmG/+^;Ptpn11^fl/fl^* mice that were similarly treated with tamoxifen served as controls.

### Chondrocyte Pellet Culture

Chondrocytes were harvested from the rib cages of newborn mouse pups as previously described [50]. Pups were either wild-type (mixed 129/FVB background) or, for SHP2 depletion experiments, *Tg(CMV-CreERT2);Ptpn11^fl/–^* on a mixed 129/FVB background. Chondrocytes from 4 to 7 pups from the same litter and with the same genotype were pooled, which provided sufficient cells for all the experimental and control pellets of an individual experiment. Chondrocytes were re-suspended in DMEM/F12 with L-Glutamine (Invitrogen), supplemented with ascorbic acid (50 µg/ml), 1% Penicillin-Streptomycin solution (Life Technologies) and 10% Fetal Bovine Serum (Omega Scientific). Five × 10^5^ cells were aliquoted into 0.5 mL eppendorf tubes, centrifuged at 500 g for 5 min, and then cultured in 400 µL of medium at 37°C and 5% CO_2_. Medium was changed every 1 to 2 days. Stock solutions of 10 mM U0126 (Sigma) dissolved in DMSO, and 1 mM 4-OHT (Sigma) dissolved in 100% EtOH were aliquoted and stored at −20°C. For MEK1/2 inhibition experiments, starting at day 2 of culture, pellets were switched to medium containing 10 µM U0126 or 0.1% DMSO (vehicle control), which was changed daily, and pellets were harvested after 1 or 3 weeks. For SHP2 depletion or 4-OHT-treatment experiments, starting at day 1 pellets were placed in medium containing 1 µM 4-OHT or 0.1% EtOH (vehicle control), and either harvested after 1 week, or treated for an additional week, switched to untreated medium for 1 week, and then harvested (after 3 weeks total in culture).

### Rna Sequencing Library Construction And Data Analysis

Two pellet cultures were pooled prior to performing RNA extraction with the RNeasy mini kit (Qiagen) following the manufacturer's recommendation. RNA extracts, which contained 200 to 1,000 ng RNA were used to prepare one barcoded cDNA library, using the TruSeq RNA Library Preparation Kit (Illumina). Three different barcoded cDNA libraries were made for each genotype, treatment group, and time point. Eight to 12 separate libraries were pooled and used to obtain 50-basepair paired-end reads per single lane of an Illumina HiSeq. Reads were aligned to the mouse genome (mm9) using Tophat [51]. The number of reads mapping to the exons of each gene were calculated using the GenomicFeatures R package [52]. A trimmed mean method (excluding the 0.5% most and least abundant transcripts) was used to calculate the Pearson's Correlation Coefficient between libraries [53]. Tests for differential gene expression were performed with edgeR [54] and *p* values were adjusted for multiple hypothesis testing. For identification of differentially expressed gene sets, only genes with an RPKM > 3, a fold change of at least 1.6, and an adjusted *p* value <0.05 were included. A Chi-squared test was performed to determine whether the overlap between differentially expressed transcripts at w1 and w3, and in *Ptpn11* cKO pellets and U1026 treated pellets, was significant.

### Histology, Fluorescence Microscopy, And Immunohistochemistry

Chondrocyte pellet cultures were fixed in 4% paraformaldehyde (PFA) for 1 hour at room temperature. Mouse vertebrae and long bones were fixed in 4% PFA overnight at 4°C and then decalcified for 14 days in 0.5 M EDTA pH 8 (Apex BioResearch Products). All specimens were then either frozen in OCT embedding media (Tissue Tek) and stored at −80°C, or were stored in 70% EtOH at 4°C and then dehydrated in graded ethanol, cleared in xylene, and embedded in paraffin following standard methods. Paraffin-embedded exostoses were obtained from individuals with MC who were previously described (Patients A-IV-8, A-IV-5 and B-IV-7 in Bowen *et al* (2011)). For histological analysis, 6 µm paraffin sections were stained with 0.1% Alcian Blue in 0.1 M HCl for 30 minutes and then counterstained with Nuclear Fast Red (Sigma) for 15 minutes. For TRAP staining, sections were incubated at 37°C for 30 min in 200 ml of 50 mM Sodium Tartrate 0.1 M sodium acetate buffer pH 5, containing 30 mg Fast Red Violet LB Salt (Sigma), and 48 mg Naphtol AS-MX phosphate (Sigma) that had been dissolved in 2 ml Formamide. For fluorescence microscopy, frozen sections were mounted in DAPI-Fluoromount (SouthernBiotech). Immunohistochemistry was performed on 6 µm paraffin sections using the Vectastain Elite ABC kit (Vector Labs) following the manufacturer's instructions and using the hybridization buffers, secondary antibodies and visualization reagents provided in the kit. To unmask antigens, tissue sections were first incubated in Citrate Buffer (10 mM citric acid, 0.05% Tween 20, pH 6.0) for 20 min at 90°C. To detect the extracellular proteins Type 10 collagen (COLX), Indian Hedgehog (IHH) and Secreted phosphoprotein 1 (SPP1), sections were also treated with 1 mg/ml Hyaluronidase (Sigma) for 1 hr at 37°C and Pepsin Solution (Diagnostic Biosystems) for 15 min at 37°C. The following antibodies and dilutions were used: mouse anti-COLX (X53; Quartett) at 1∶100, rabbit anti-FRA1 (D80B4; Cell Signaling) at 1∶400; rabbit anti-IHH (N-term; Abgent) at 1∶100; rabbit anti-SPP1 (from the laboratory of Dr. Dick Heinegard, Lund University) at 1∶3,000; rabbit anti-ERK1/2 (137F5; Cell Signaling) at 1∶400; rabbit anti-CD31 (ab28364; Abcam) at 1∶40; and rabbit anti-phospho-SMAD1/5/8 (Cell Signaling Technology) at 1∶400. Primary antibodies were incubated with the tissue sections overnight at 4°C.

### Quantitative Reverse-Transcriptase Pcr (qrt-Pcr)

Total RNA (500 ng) extracted from the pellet cultures was used to synthesize cDNA with the QuantiTect Reverse Transcription Kit (Qiagen) in a 20 µl reaction volume. qRT-PCR was performed using SYBR Green JumpStart Taq ReadyMix (Sigma) and 1 µl of cDNA per reaction. Reactions were performed in triplicate. Changes in transcript abundance were calculated using the ddCt method [55] with β-actin (*Actb*) as the reference transcript. *Actb* was selected as a reference transcript because our RNA-seq data indicated that its abundance was not significantly affected by SHP2 depletion, 4-OHT treatment or U0126 treatment. Gene-specific primer pairs are listed in Table S6.

### Immunoblotting

Individual chondrocyte pellet cultures were homogenized in 60 µL of Pierce IP Lysis Buffer (Thermo Scientific) with freshly added Halt Protease and Phosphatase Inhibitor (Thermo Scientific). Twenty μL of lysate was added to 7.5 µL of NuPAGE LDS Sample Buffer and 3 µl NuPAGE Reducing Agent, heated to 70°C for 10 minutes, and loaded onto a 4–20% NuPAGE Bis-Tris Mini Gel (Life Technologies). Following transfer to Invitrolon PVDF membranes (Life Technologies) using standard procedures, immunodetection was performed using the Western Breeze Chemiluminescent Immunodetection kit (Life Technologies). The following primary antibodies were used: mouse anti-SHP2 (M163; Abcam), rabbit anti-phospho-ERK1/2 (Thr202/Tyr204; D13.14.4E; Cell Signaling), rabbit anti-ERK1/2 (137F5; Cell Signaling), and rabbit anti-phospho-AKT (Ser473;D9E; Cell Signaling). All primary antibodies were diluted to 1∶1000 and incubated overnight at 4°C.

## Supporting Information

Figure S1 Correlation between libraries from the same time point. Scatterplots comparing the number of reads mapping to each gene in RNA-seq libraries prepared from pellets harvested after 1 week in culture. Each circle represents an individual gene. A: Comparison of two representative RNA-seq libraries prepared from pellets that were established at the same time from the same litter of mice. B: Comparison of two representative RNA-seq libraries prepared on different days from pellets that were independently established from different mouse litters. Pellets in set A were untreated. Pellets in set B were treated with 0.1% DMSO and were used as vehicle control pellets in our MEK1/2-inhibition experiment. The Pearson correlation coefficient (R^2^), calculated using the trimmed-mean method, is indicated on each plot. (JPG)

Figure S2 Vehicle treatment alone does not significantly alter chondrocyte maturation in pellet cultures. Heatmap showing the fold change in transcript abundance over time (from w1 to w3) in three independent pellet culture experiments, for a set of transcripts associated with the proliferative zone (PZ) or late-hypertrophic zone (late-HZ). A  =  untreated pellets. B  =  pellets treated with 0.1% DMSO as a vehicle control in our MEK1/2-inhibition experiments. C  =  pellets treated with 0.1% EtOH as a vehicle control in our 4-OHT-treatment experiments. (JPG)

Figure S3 Using the *mTmG* reporter to monitor CreER mediated recombination in pellet cultures. Merged images of GFP, RFP and DAPI channels after daily 4-OHT treatment of chondrocytes pellet cultures established from mice carrying the *mTmG* reporter allele. The *mTmG* reporter initially drives expression of *Tomato Fluorescent Protein* (*TFP*), but after CreER-mediated recombination, it drives the expression of *Green Fluorescent Protein* (*GFP*). The number of green cells (recombined) and red cells (un-recombined) were counted in multiple tissue sections from pellets cultured for 3, 4, 5 or 7 days. The average percentage of recombined cells at each time point is indicated. Based on the half-life of the TFP protein, some TFP will still be present in cells with a relatively recent recombination event, and these cells will appear yellow. (JPG)

Figure S4 Overlap between transcripts that change in abundance after SHP2 depletion, 4-OHT treatment, or U0126 treatment. A: Heat map of all transcripts that changed in abundance after SHP2 depletion, 4-OHT treatment or U0126 treatment, at either w1 or w3. B: Venn diagrams showing the overlap of transcripts that decrease (blue) or increase (red) in abundance, at w1 or w3, in U0126-treated (left circle), *Ptpn11*-cKO (right circle) and 4-OHT-treated (lower circle) pellets. (JPG)

Figure S5 Overlap between transcripts that changed in abundance at w1 and w3. Venn diagrams showing the overlap between transcripts that increased (red) or decreased (blue) in abundance after SHP2 depletion (A) or U1026 treatment (B) at w1 and w3. (JPG)

Figure S6 Gene Process Networks enriched in differentially expressed genes. List of enriched Gene Process Networks, identified using the MetaCore software suite from GeneGo Inc, in the list of transcripts that increased (red) or decreased (blue) in abundance after U0126-treatment, SHP2 depletion, or 4-OHT treatment, at w1 or w3. The list of transcripts included in each network is indicated. Only networks with a MetaCore p value less than 1×10^-6^ are shown. (JPG)

Figure S7 Identification of transcripts associated with chondrocyte maturation that changed in abundance after MEK1/2 inhibition or SHP2 depletion. Heatmaps for a subset of transcripts identified in our “upper zones” (A) or “lower zones” (B) transcript sets that significantly changed in abundance at w3 after either MEK1/2 inhibition or SHP2 depletion. Transcripts were selected based on their known roles in processes that are relevant to proliferative chondrocytes (e.g., matrix synthesis) or late-hypertrophic chondrocytes (e.g., matrix degradation). Some, but not all, of the “lower zones” transcripts that decreased in abundance after SHP2 depletion also decreased in abundance after 4-OHT treatment of wild-type pellets. The increase in the abundance of transcripts encoded by the mitochondrial genome may be a result of the increase in the number of mitochondria per cell due to the increase in chondrocyte cell size following SHP2 depletion or MEK1/2 inhibition. (JPG)

Figure S8 Overlap between transcripts that changed in abundance following SHP2 depletion and MEK1/2 inhibition. Venn diagrams showing the overlap between transcripts that increased (red) or decreased (blue) in abundance after SHP2 depletion or U1026 treatment at w1 (A) and w3 (B). (JPG)

Figure S9 qRT-PCR validation of transcripts that changed in abundance following MEK1/2 inhibition at w3. Graph showing expression, relative to *beta-actin*, of selected genes in vehicle treated pellets (black) and U0126-treated pellets (grey). For each gene, the expression levels were normalized such that the expression level in vehicle treated pellets equals 1. Error bars indicate standard deviation. Asterisk indicates a significant (p<0.05) difference between Ctrl and U0126-treated pellets. (JPG)

Figure S10 MEK1/2- and SHP2-dependent transcription factors. Heatmap showing all 57 transcription factors that were differentially expressed at w3 in *Ptpn11*-cKO or U0126-treated pellets. For comparison purposes, the fold change at w1 is also shown, and the fold change in 4-OHT treated pellets is shown. (JPG)

Figure S11 Phenotypic range observed in Col2a1-cKO vertebral growth plates. Tissue sections of lumbar vertebral growth plates from *Col2a1-CreER; Rosa26^mTmG^* mice that were heterozygous (Ctrl mice) or homozygous (Col2a1-cKO mice) for a conditional *Ptpn11* allele and had been administered tamoxifen for 5 days, starting at w1, and sacrificed at week 3, 7 or 10. Tissue sections were stained with Alcian Blue and Nuclear Fast Red. In Col2a1-cKO mice, the vertebral growth plates are substantially enlarged and disorganized at week 7 and 10, with osteophyte-like lateral outgrowths, and ectopic cartilage islands below the growth plate (arrows). By week 10, some growth plates had been completely resorbed (asterisks). Scale bar  =  250 µm. (JPG)

Figure S12 Osteoclast recruitment and vascular invasion are not affected in Col2a1-cKO vertebral growth plates. Tissue sections of lumbar vertebral growth plates from Ctrl and Col2a1-cKO mice at w10. A: Tartrate-Resistant Acid Phosphatase (TRAP) staining to mark osteoclasts at the bone-cartilage junction (arrows). Sections are counterstained with Alcian Blue. B: Immunohistochemical staining for CD31, to mark endothelial cells. Sections are counterstained with hematoxylin. Scale bar  =  100 µm. (JPG)

Figure S13 Ectopic cartilage is not observed in Fsp1-cKO mice at week 3. A: Radiographs of the forelimbs and hindlimbs of 3-week-old Ctrl and Fsp1-cKO mice. No noticeable skeletal abnormalities are observed in Fsp1-cKO mice at this time point. B: Alcian Blue and Nuclear Fast Red stained sections of the radius and ulna of Ctrl and Fsp1-cKO mice at week 3 (upper) and week 20 (lower). Note the presence of ectopic cartilage in the interosseous membrane between radius and ulna at week 20 in Fsp1-cKO mice (arrow) but not at week 3. Scale bars: A  =  1 mm; B  =  100 µm. (JPG)

Figure S14 Ectopic cartilage is observed on the surface of the femur and tibia of Fsp1-cKO mice. A-B: Alcian Blue and Nuclear Fast Red stained femur (A) and tibia (B) sections from 20-week-old Ctrl and Fsp1-cKO mice. Note the presence of ectopic chondrocytes (arrow, A) on the surface of the diaphyseal cortical bone of the femur, and the cartilaginous outgrowth (arrow, B) on the articular surface of the tibia, adjacent to the attachment site of the anterior cruciate ligament (ACL). C: Merged fluorescent images showing recombined cells (green), un-recombined cells (red) and nuclei (blue) in sections from 20-week-old Ctrl mice (*Fsp1-Cre;Ptpn11^fl/+^;Rosa26^mTmG^*) and Fsp1-cKO mice (*Fsp1-Cre;Ptpn11^fl/fl^;Rosa26^mTmG^*). In Ctrl mice, evidence of *Fsp1-Cre*-mediated recombination (arrow) is observed in a few scattered fibroblast-like cells at the attachment site of the posterior cruciate ligament (PCL). In Fsp1-cKO mice, cells with a history of *Fsp1-Cre*-mediated recombination are located at the ligament attachment site and have acquired the round morphology of chondrocytes (arrow). Scale bars  =  100 µm. (JPG)

Table S1 Number of reads and mapping statistics for each library. (PDF)

Table S2 Previous studies identifying transcripts that are associated with a specific chondrocyte maturation stage. (PDF)

Table S3 Effect of SHP2 depletion or MEK1/2 inhibition on markers of chondrocyte maturation zones. (PDF)

Table S4 Previous studies identifying transcription factors involved in chondrocyte maturation and/or terminal differentiation. (PDF)

Table S5 Genes regulated by MEK1/2-dependent transcription factors. (PDF)

Table S6 Transcript-specific primers used for q-RT-PCR. (PDF)

Dataset S1 Transcripts detected by RNA-seq on chondrocyte pellet cultures. Dataset of RPKM expression values for all transcripts detected in wild-type pellet cultures, as well as the fold change for each transcript that significantly changed in abundance following SHP2 depletion, MEK1/2 inhibition or 4-OHT treatment. (XLSX)
