# Supplementary figures and images for: SHP2 Regulates Chondrocyte Terminal Differentiation, Growth Plate Architecture and Skeletal Cell Fates
Source: PLoS Genet. 2014 May 29;10(5):e1004364. doi: 10.1371/journal.pgen.1004364 (PMC4038465; doi:10.1371/journal.pgen.1004364)

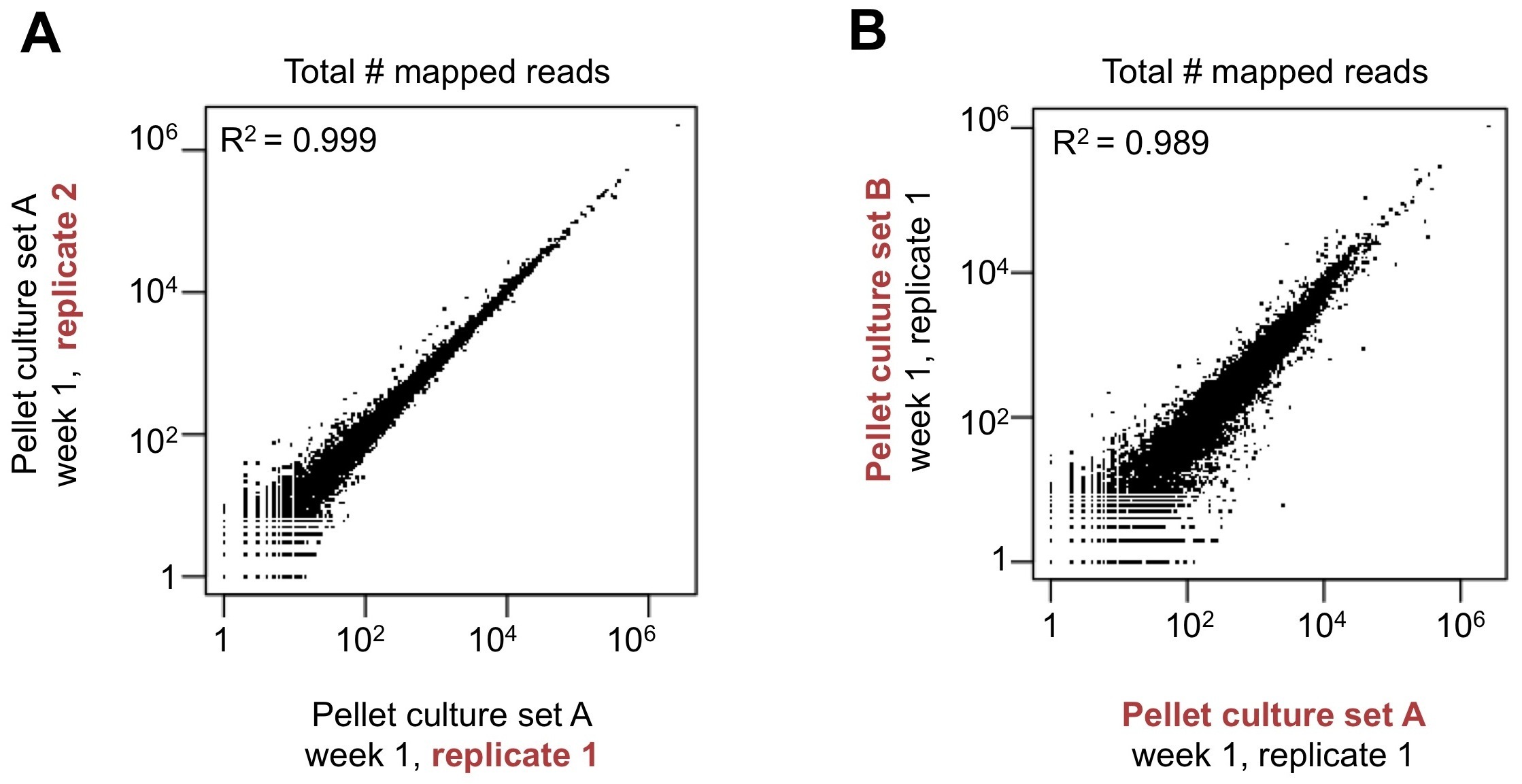

Supplement: Figure S1 — (JPG) [file pgen.1004364.s001.jpg]

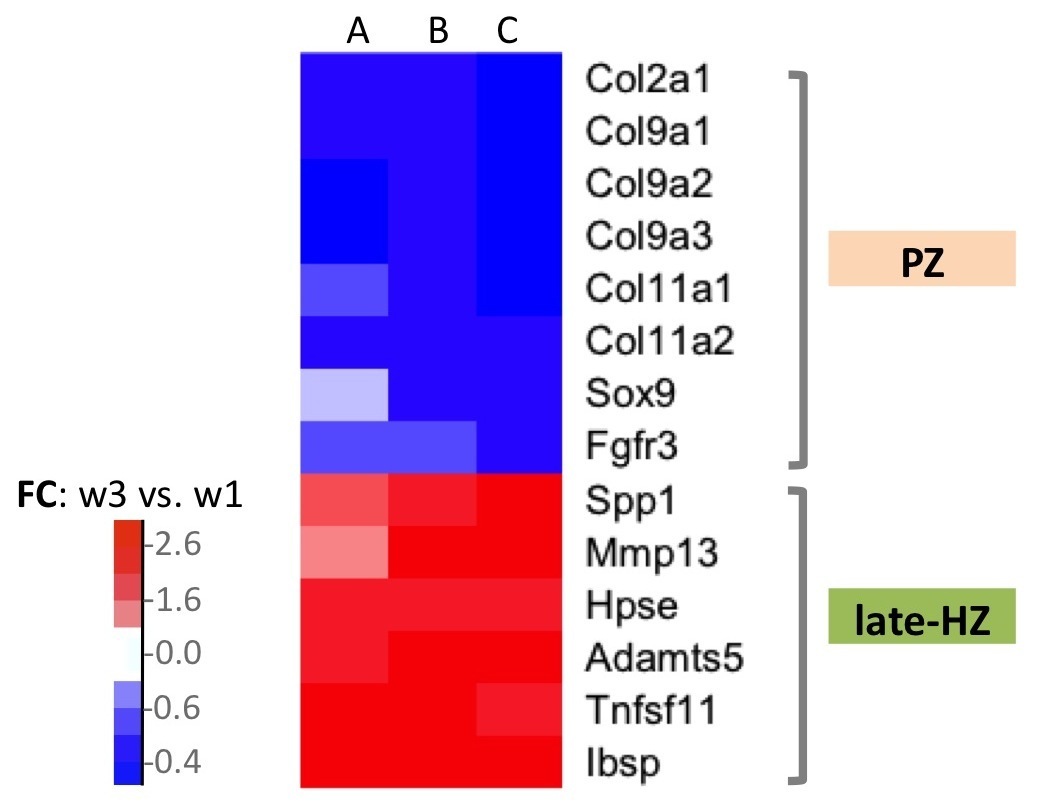

Supplement: Figure S2 — (JPG) [file pgen.1004364.s002.jpg]

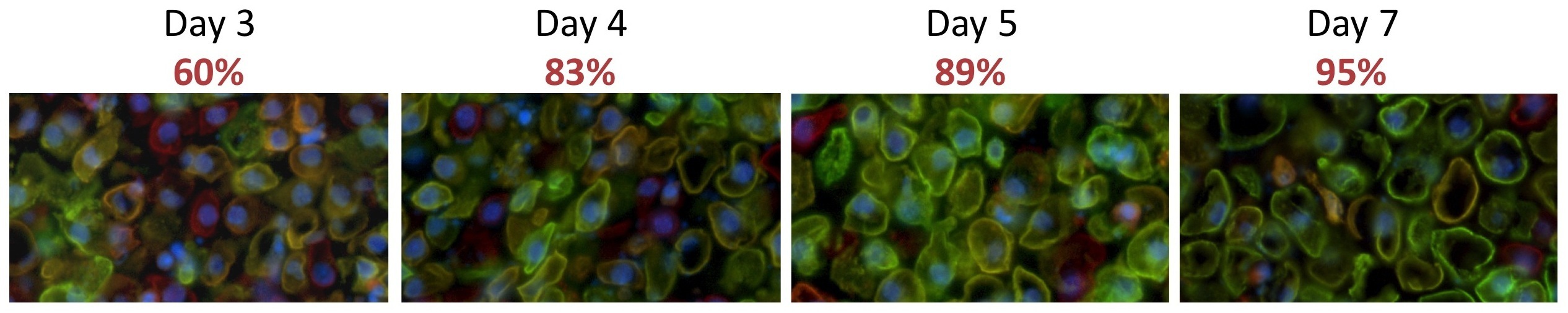

Supplement: Figure S3 — (JPG) [file pgen.1004364.s003.jpg]

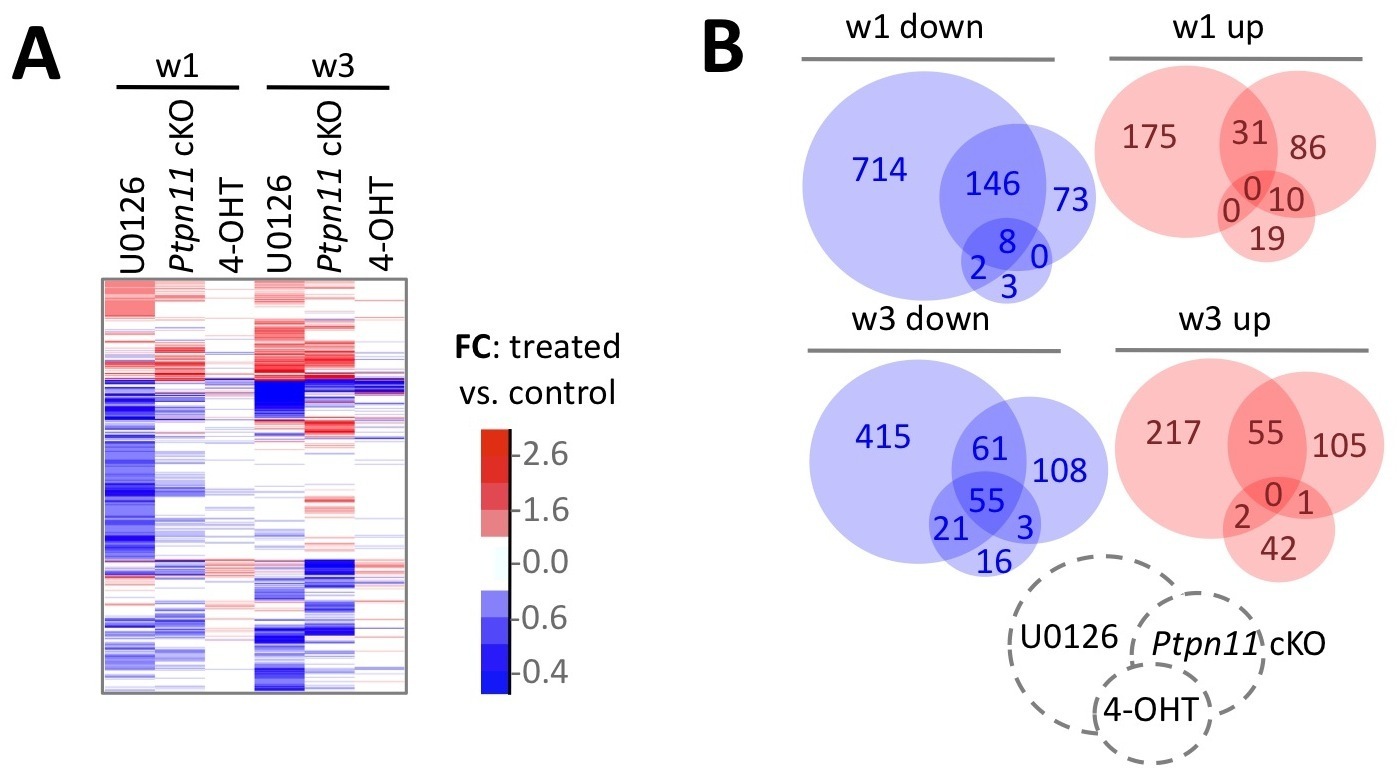

Supplement: Figure S4 — (JPG) [file pgen.1004364.s004.jpg]

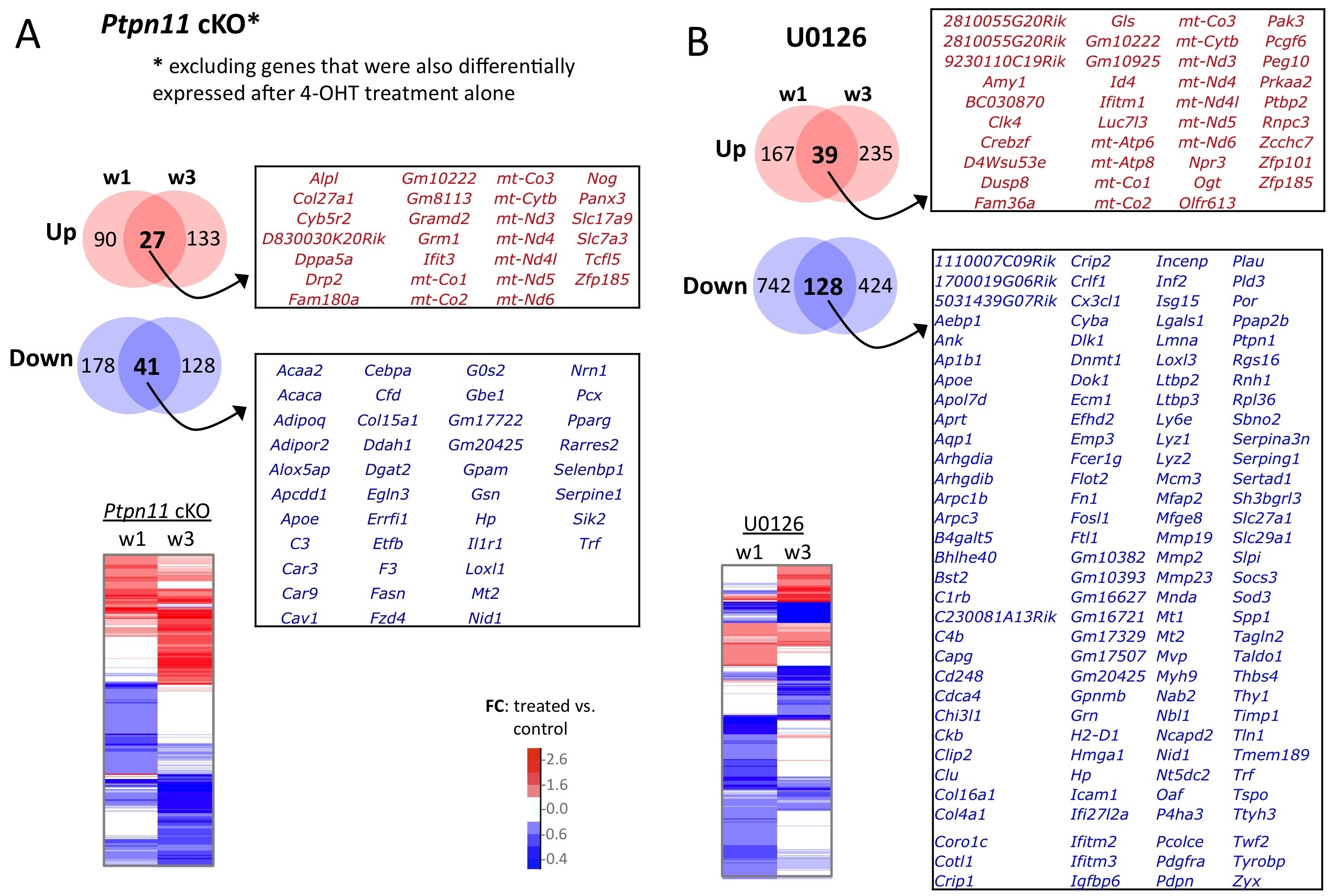

Supplement: Figure S5 — (JPG) [file pgen.1004364.s005.jpg]

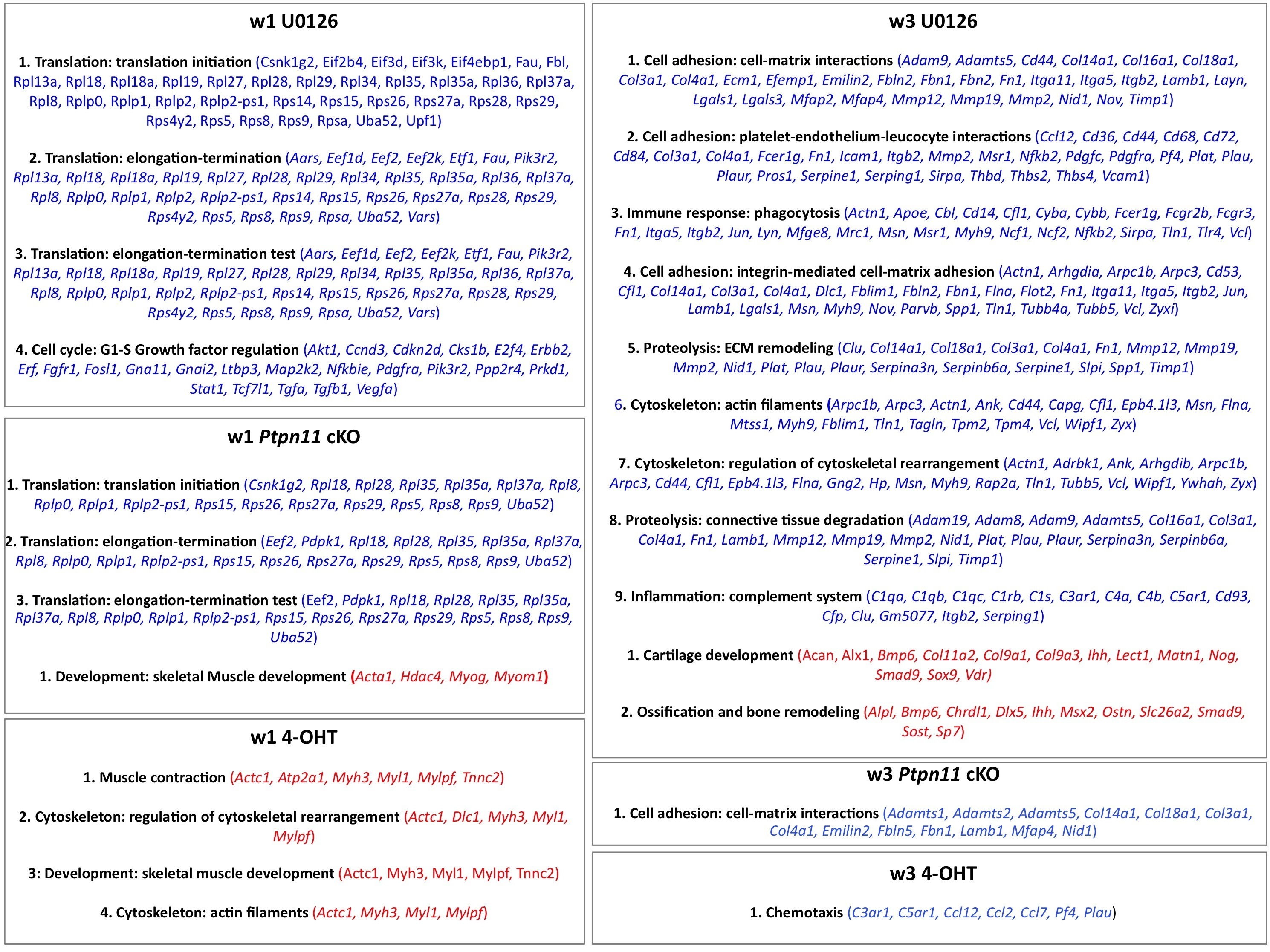

Supplement: Figure S6 — (JPG) [file pgen.1004364.s006.jpg]

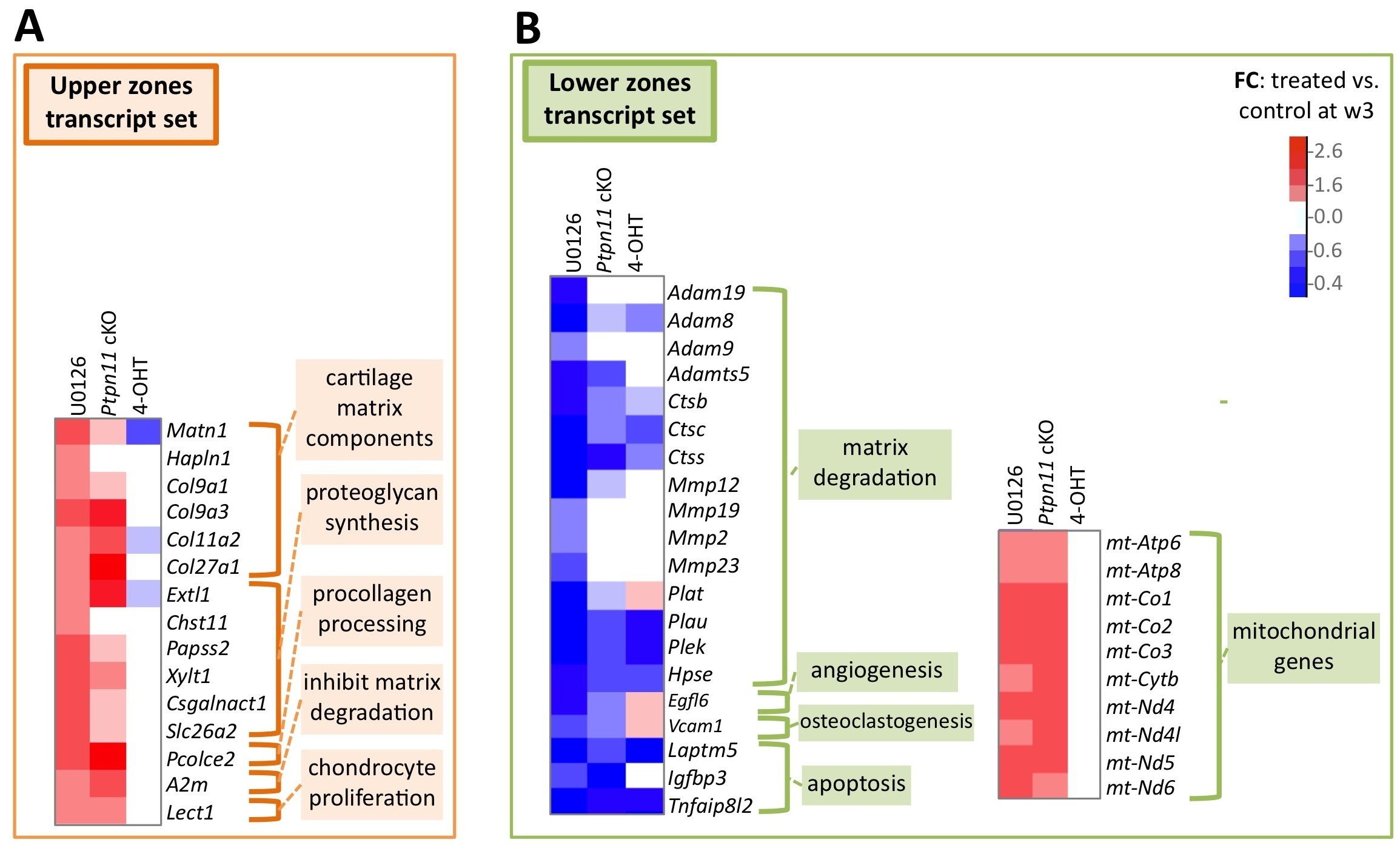

Supplement: Figure S7 — (JPG) [file pgen.1004364.s007.jpg]

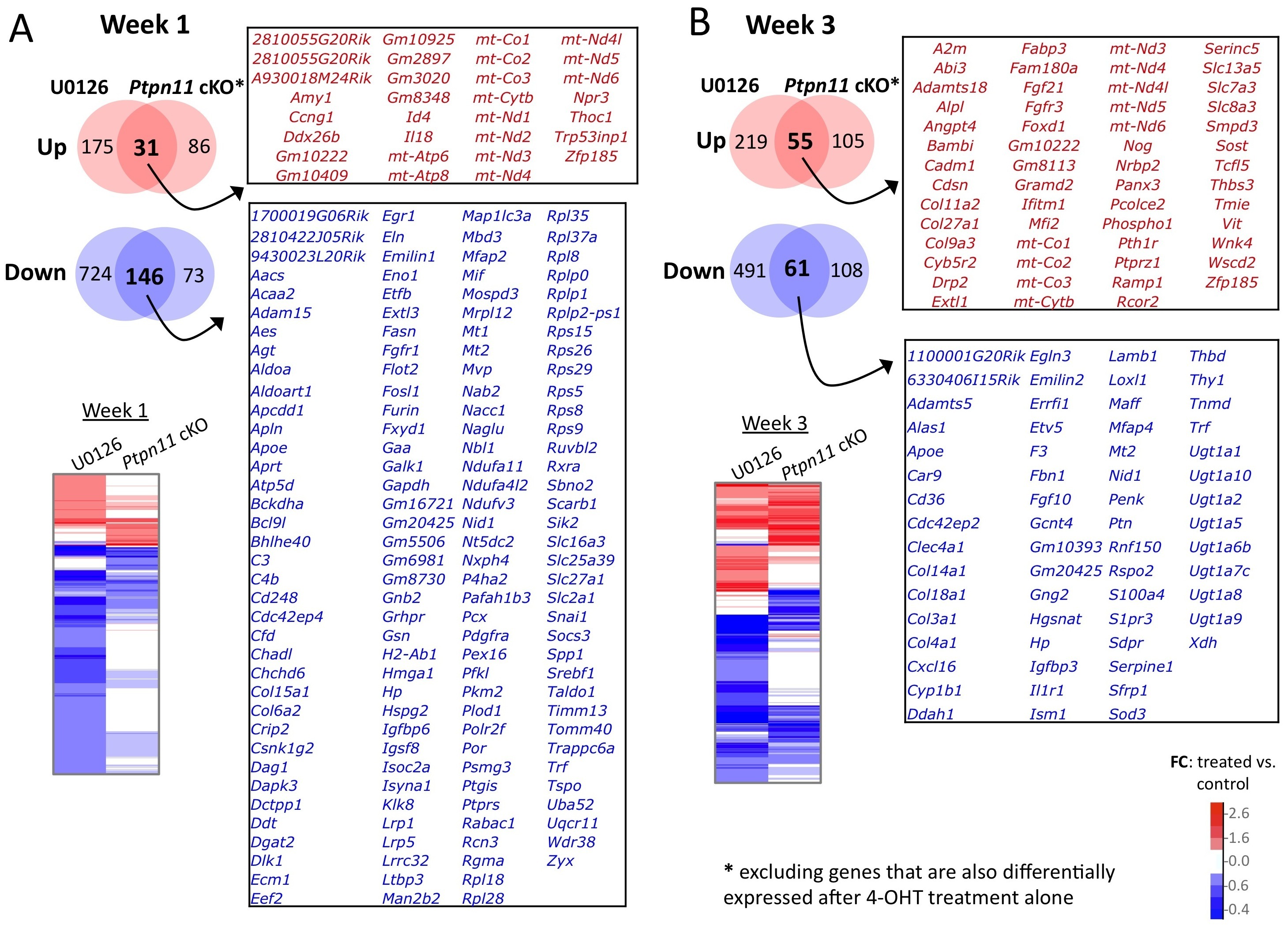

Supplement: Figure S8 — (JPG) [file pgen.1004364.s008.jpg]

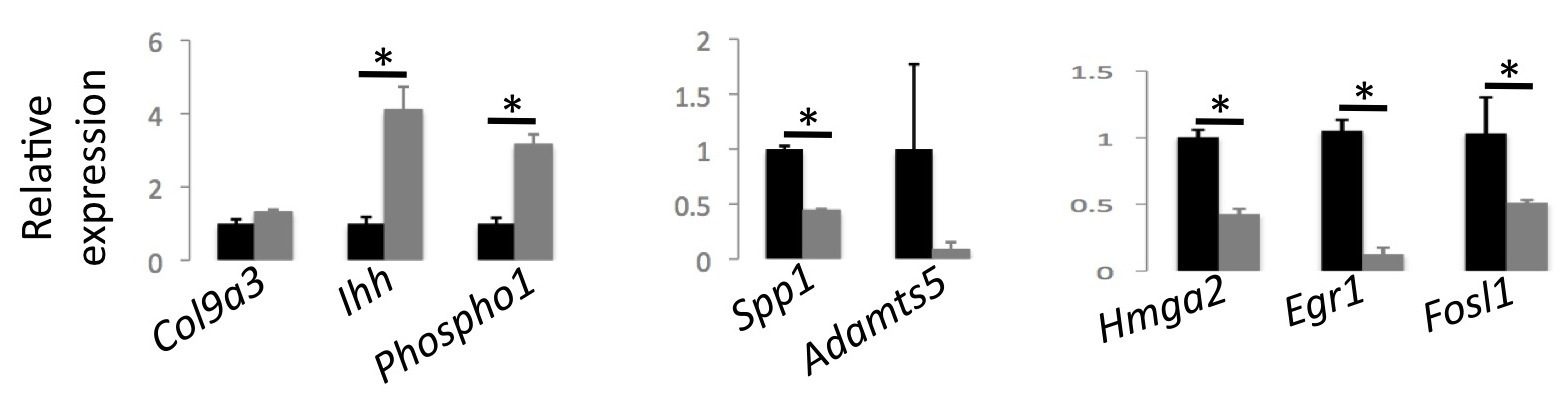

Supplement: Figure S9 — (JPG) [file pgen.1004364.s009.jpg]

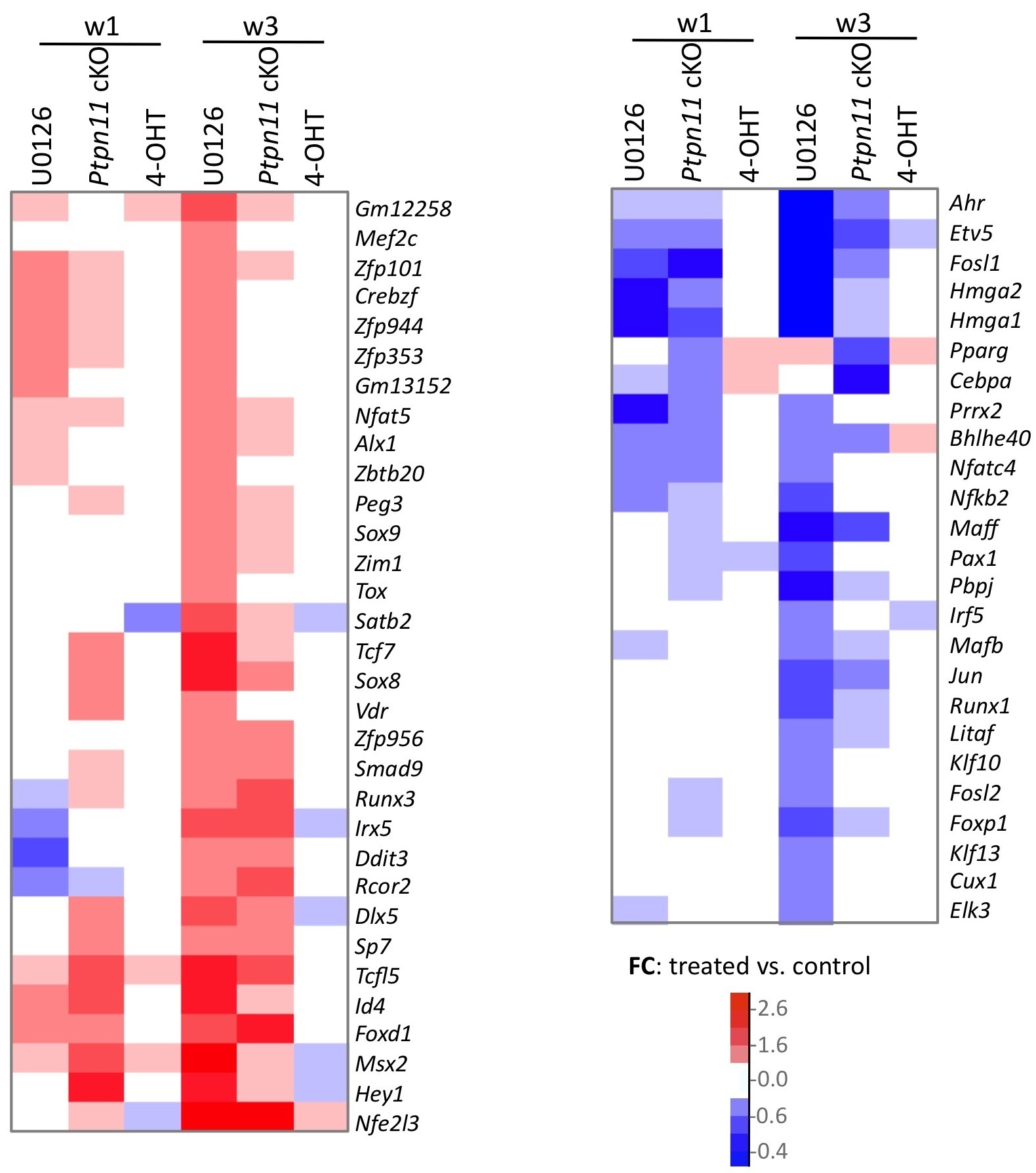

Supplement: Figure S10 — (JPG) [file pgen.1004364.s010.jpg]

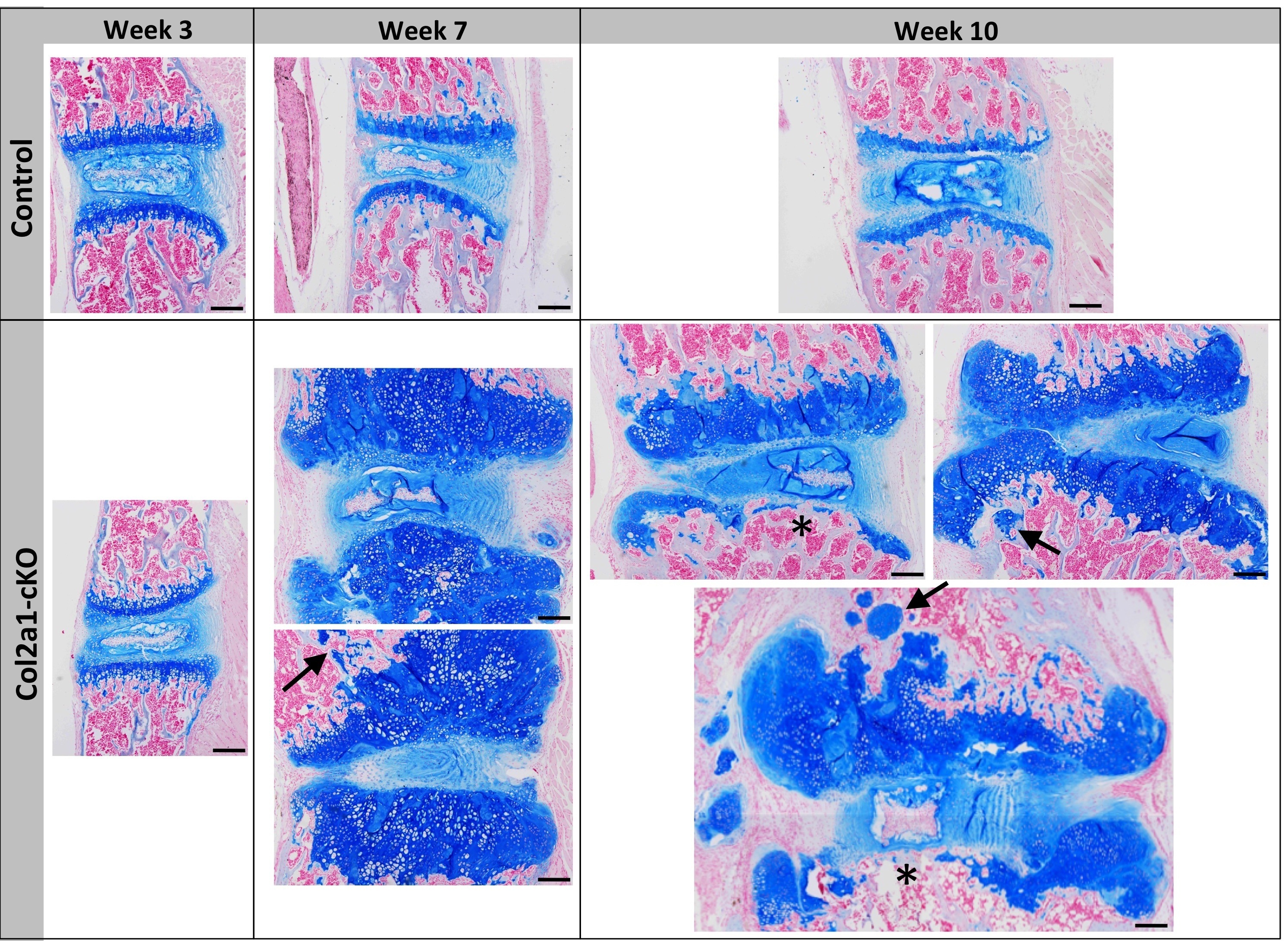

Supplement: Figure S11 — (JPG) [file pgen.1004364.s011.jpg]

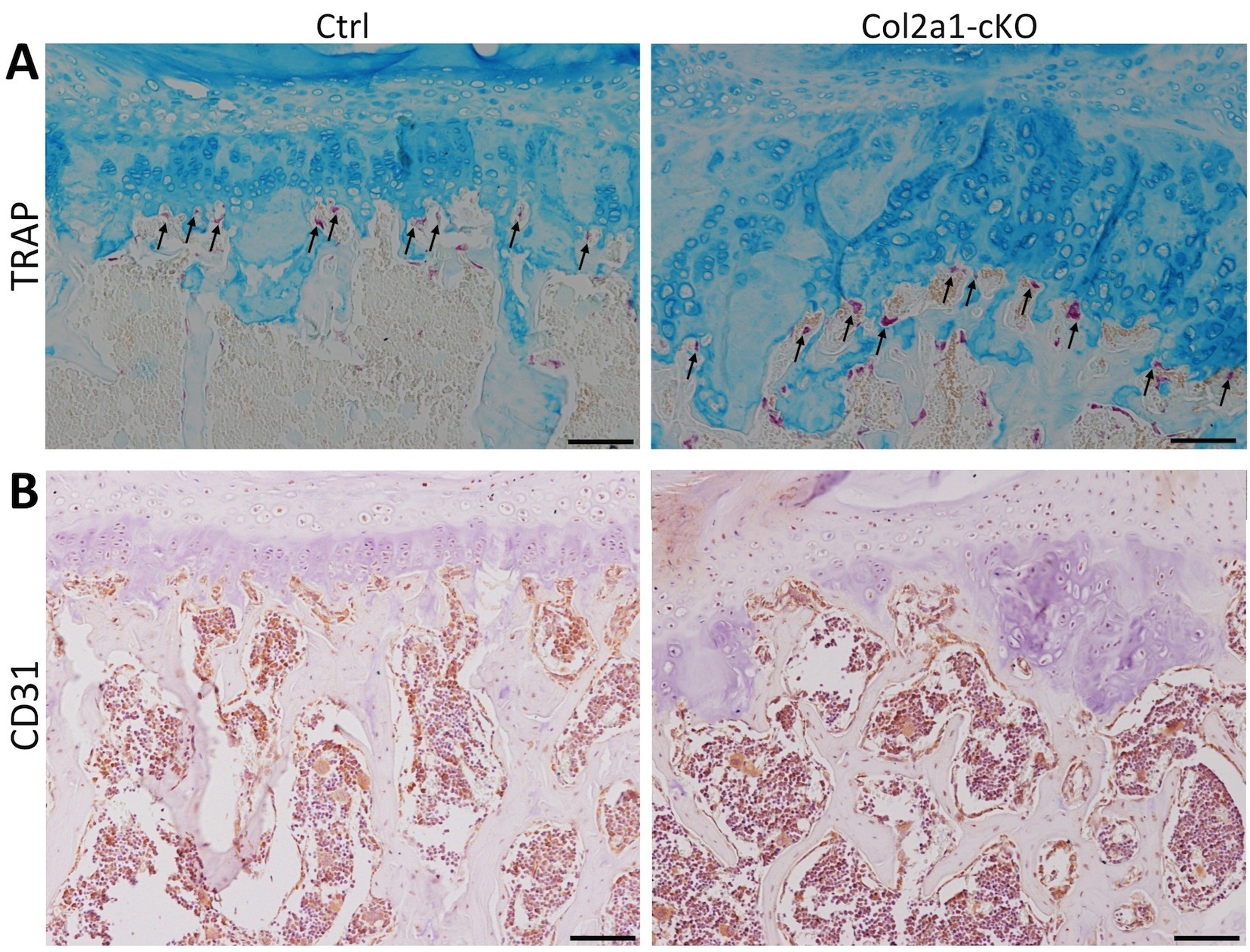

Supplement: Figure S12 — (JPG) [file pgen.1004364.s012.jpg]

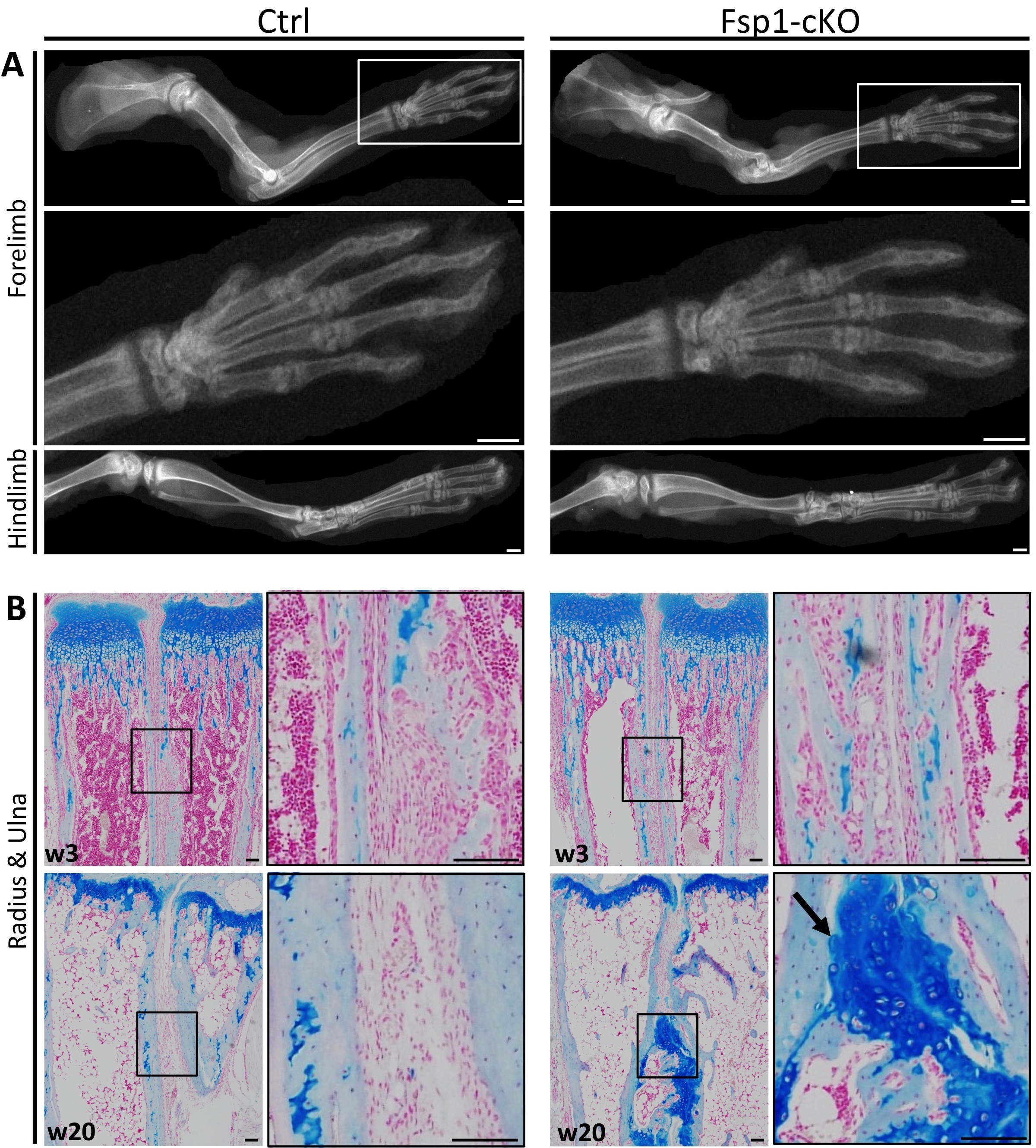

Supplement: Figure S13 — (JPG) [file pgen.1004364.s013.jpg]

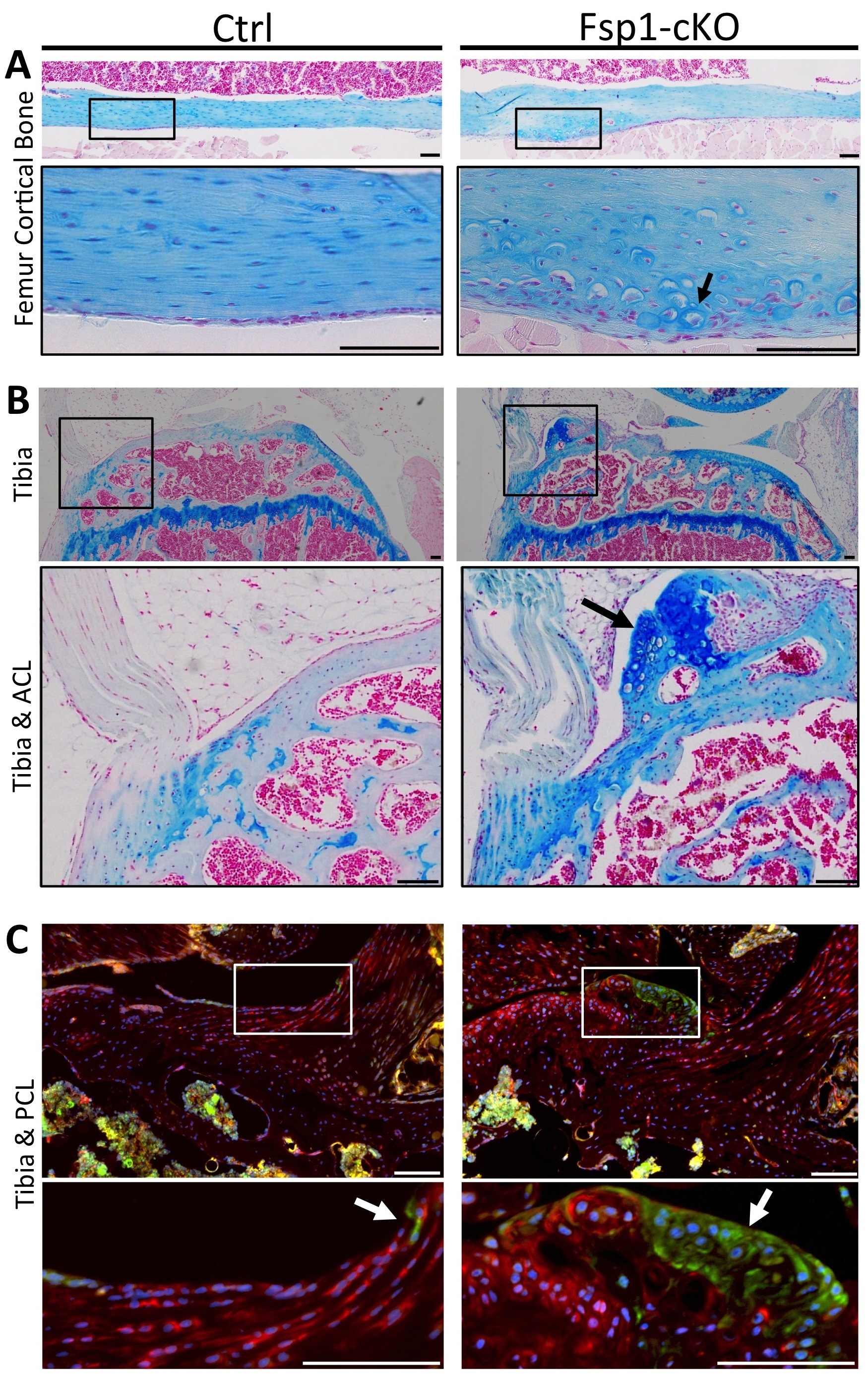

Supplement: Figure S14 — (JPG) [file pgen.1004364.s014.jpg]
