# Supplementary material for: SHP2 Regulates Chondrocyte Terminal Differentiation, Growth Plate Architecture and Skeletal Cell Fates
Source: PLoS Genet. 2014 May 29;10(5):e1004364. doi: 10.1371/journal.pgen.1004364 (PMC4038465; doi:10.1371/journal.pgen.1004364)
Supplement: Table S1 — (PDF) [file pgen.1004364.s015.pdf]

Mouse litter	Genotype & time point	Replicate	Total # reads (50bp paired end)	% mapped
**A**	**Day 4**	1	24,958,041	96%
	Wild-type	2	10,908,661	97%
		3	13,805,624	97%
	**Week 1**	1	13,367,197	95%
	Wild-type	2	13,300,200	98%
		3	9,673,123	97%
	**Week 2**	1	13,234,707	96%
	Wild-type	2	9,378,957	96%
		3	10,341,598	98%
	**Week 3**	1	11,077,264	96%
	Wild-type	2	10,969,002	97%
		3	13,946,829	97%
**B**	**Week 1**, 4-OHT	1	16,566,070	96%
	Wild-type	2	17,246,647	96%
		3	16,205,375	96%
	**Week 1**, untreated	1	17,057,605	97%
	Wild-type	2	23,336,815	97%
		3	25,856,434	97%
	**Week 3**, 4-OHT	1	20,776,870	95%
	Wild-type	2	16,113,528	96%
		3	14,954,606	85%
	**Week 3**, untreated	1	15,769,721	97%
	Wild-type	2	14,294,479	95%
		3	20,438,780	97%
**C**	**Week 1**, 4-OHT	1	12,110,000	93%
	Ptpn11 –/fl; CMV-CreERT2	2	9,657,059	94%
		3	9,507,599	96%
	**Week 1**, untreated	1	25,954,789	95%
	Ptpn11 –/fl; CMV-CreERT2	2	12,766,566	97%
		3	7,648,448	86%
	**Week 3**, 4-OHT	1	31,478,241	97%
	Ptpn11 –/fl; CMV-CreERT2	2	40,187,879	96%
		3	31,181,967	96%
	**Week 3**, untreated	1	26,929,091	96%
	Ptpn11 –/fl; CMV-CreERT2	2	18,316,729	93%
		3	21,570,699	94%
**D**	**Week 1**	1	14,138,829	97%
	U0126	2	12,634,668	96%
		3	13,823,998	96%
	**Week 1**	1	12,165,140	97%
	DMSO control	2	11,748,157	96%
		3	12,558,375	97%
	**Week 3**	1	12,765,004	93%
	U0126	2	11,862,249	97%
		3	13,849,488	95%
	**Week 3**	1	13,018,137	94%
	DMSO control	2	12,150,097	93%
		3	12,601,300	96%
