# Supplementary material for: SHP2 Regulates Chondrocyte Terminal Differentiation, Growth Plate Architecture and Skeletal Cell Fates
Source: PLoS Genet. 2014 May 29;10(5):e1004364. doi: 10.1371/journal.pgen.1004364 (PMC4038465; doi:10.1371/journal.pgen.1004364)
Supplement: Table S2 — (PDF) [file pgen.1004364.s016.pdf]

Growth plate zone	Transcript	Reference
**PZ**	*Col2a1*	[56]
	Col9a1	[56]
	Col9a2	[56]
	Col9a3	[56]
	Col11a1	[56]
	Col11a2	[56]
	Sox9	[38]
	Fgfr3	[57]
**Pre-HZ**	*Pth1r*	[58]
	Ihh	[58]
	Panx3	[59]
	Sp7	[60]
**Early-HZ**	*Col10a1*	[38]
	Phospho1	[61]
	Bmp6	[62]
	Alpl	[63]
**Late-HZ**	*Spp1*	[38]
	Mmp13	[38]
	Hpse	[64]
	Adamts5	[65]
	Adamts1	[66]
	Tnfsf11	[67]
	Ibsp	[68]

Additional References

56. Keene DR, Oxford JT, Morris NP (1995) Ultrastructural localization of collagen types II, IX, and XI in the growth plate of human rib and fetal bovine epiphyseal cartilage: type XI collagen is restricted to thin fibrils. Journal of Histochemistry & Cytochemistry 43: 967–979. doi:10.1177/43.10.7560887.

57. Naski MC, Colvin JS, Coffin JD, Ornitz DM (1998) Repression of hedgehog signaling and BMP4 expression in growth plate cartilage by fibroblast growth factor receptor 3. Development 125: 4977–4988.

58. Shung C-Y, Ota S, Zhou Z-Q, Keene DR, Hurlin PJ (2012) Disruption of a Sox9- -catenin circuit by mutant Fgfr3 in thanatophoric dysplasia type II. Human Molecular Genetics 21: 4628–4644. doi:10.1093/hmg/dds305.

59. Iwamoto T, Nakamura T, Doyle A, Ishikawa M, de Vega S, et al. (2010) Pannexin 3 Regulates Intracellular ATP/cAMP Levels and Promotes Chondrocyte Differentiation. Journal of Biological Chemistry 285: 18948–18958. doi:10.1074/jbc.M110.127027.

60. Nishimura R, Wakabayashi M, Hata K, Matsubara T, Honma S, et al. (2012) Osterix Regulates Calcification and Degradation of Chondrogenic Matrices through Matrix Metalloproteinase 13 (MMP13) Expression in Association with Transcription Factor Runx2 during Endochondral Ossification. Journal of Biological Chemistry 287: 33179–33190. doi:10.1074/jbc.M111.337063.

61. Houston B, Stewart AJ, Farquharson C (2004) PHOSPHO1-A novel phosphatase specifically expressed at sites of mineralisation in bone and cartilage. Bone 34: 629–637. doi:10.1016/j.bone.2003.12.023.

62. Vu TH, Shipley JM, Bergers G, Berger JE, Helms JA, et al. (1998) MMP-9/gelatinase B is a key regulator of growth plate angiogenesis and apoptosis of hypertrophic chondrocytes. Cell 93: 411–422.

63. Hu H (2004) Sequential roles of Hedgehog and Wnt signaling in osteoblast development. Development 132: 49–60. doi:10.1242/dev.01564.

64. Brown AJ, Alicknavitch M, D'Souza SS, Daikoku T, Kirn-Safran CB, et al. (2008) Heparanase expression and activity influences chondrogenic and osteogenic processes during endochondral bone formation. Bone 43: 689–699. doi:10.1016/j.bone.2008.05.022.

65. Makihira S (2003) Thyroid Hormone Enhances Aggrecanase-2/ADAM-TS5 Expression and Proteoglycan Degradation in Growth Plate Cartilage. Endocrinology 144: 2480–2488. doi:10.1210/en.2002-220746.

66. Little CB, Mittaz L, Belluoccio D, Rogerson FM, Campbell IK, et al. (2005) ADAMTS-1-knockout mice do not exhibit abnormalities in aggrecan turnover in vitro or in vivo. Arthritis Rheum 52: 1461–1472. doi:10.1002/art.21022.

67. Silvestrini G, Ballanti P, Patacchioli F, Leopizzi M, Gualtieri N, et al. (2005) Detection of osteoprotegerin (OPG) and its ligand (RANKL) mRNA and protein in femur and tibia of the rat. J Mol Histol 36: 59–67. doi:10.1007/s10735-004-3839-1.

68. Chen J, Shapiro HS, Sodek J (2009) Developmental expression of bone sialoprotein mRNA in rat mineralized connective tissues. Journal of Bone and Mineral Research 7: 987–997. doi:10.1002/jbmr.5650070816.
