# Supplementary material for: SHP2 Regulates Chondrocyte Terminal Differentiation, Growth Plate Architecture and Skeletal Cell Fates
Source: PLoS Genet. 2014 May 29;10(5):e1004364. doi: 10.1371/journal.pgen.1004364 (PMC4038465; doi:10.1371/journal.pgen.1004364)
Supplement: Table S3 — (PDF) [file pgen.1004364.s017.pdf]

| GP zone | Gene | MEK1/2 inhibition | | SHP2 depletion | | 4-OHT treatment | |
|---|---|---|---|---|---|---|---|
| | | FC* | P value | FC* | P value | FC* | P value |
| **PZ** | *Sox9* | 2.05 | 2.4E-03 | 1.36 | 7.7E-01 | 1.13 | 1.0E+00 |
| | *Col9a1* | 1.76 | 3.3E-02 | 1.46 | 5.2E-01 | 0.99 | 1.0E+00 |
| | *Fgfr3* | 2.74 | 1.4E-05 | 2.35 | 7.7E-03 | 0.82 | 1.0E+00 |
| | *Acan* | 2.43 | 1.4E-04 | 1.32 | 7.7E-01 | 1.06 | 1.0E+00 |
| **Pre-HZ** | *Pth1r* | 2.08 | 5.0E-03 | 2.61 | 1.5E-03 | 0.82 | 1.0E+00 |
| | *Ihh* | 3.92 | 3.1E-09 | 6.27 | 7.6E-11 | 0.71 | 4.5E-01 |
| | *Panx3* | 3.42 | 1.6E-08 | 5.05 | 5.5E-09 | 0.77 | 7.0E-01 |
| | *Sp7* | 2.04 | 4.4E-03 | 1.65 | 2.5E-01 | 0.92 | 1.0E+00 |
| **Early-HZ** | *Col10a1* | 1.69 | 5.3E-02 | 1.66 | 2.5E-01 | 0.67 | 1.3E-01 |
| | *Phospho1* | 2.85 | 8.3E-06 | 2.35 | 8.5E-03 | 0.93 | 1.0E+00 |
| | *Bmp6* | 2.41 | 2.4E-04 | 2.06 | 4.1E-02 | 0.79 | 1.0E+00 |
| | *Alpl* | 1.88 | 1.9E-02 | 2.88 | 3.2E-04 | 0.86 | 1.0E+00 |
| **Late-HZ** | *Adamts1* | 0.71 | 3.5E-01 | 0.43 | 1.6E-02 | 1.28 | 8.8E-01 |
| | *Adamts5* | 0.30 | 1.1E-07 | 0.47 | 2.9E-02 | 1.10 | 1.0E+00 |
| | *Spp1* | 0.13 | 1.5E-22 | 0.88 | 1.0E+00 | 0.72 | 3.4E-01 |
| | *Hpse* | 0.34 | 1.8E-06 | 0.39 | 4.5E-03 | 0.43 | 2.9E-06 |

* FC = Fold change when comparing treated pellets to control pellets at w3
