# Supplementary material for: SHP2 Regulates Chondrocyte Terminal Differentiation, Growth Plate Architecture and Skeletal Cell Fates
Source: PLoS Genet. 2014 May 29;10(5):e1004364. doi: 10.1371/journal.pgen.1004364 (PMC4038465; doi:10.1371/journal.pgen.1004364)
Supplement: Table S4 — (PDF) [file pgen.1004364.s018.pdf]

Transcription factor	Role in the growth plate	Reference
Mef2c<br>Tcf7<br>Dlx5<br>Msx2	Promotes hypertrophy	[69]<br>[70]<br>[41]<br>[40]
Foxp1	Inhibits hypertrophy	[71]
Hey1<br>Sox9<br>Dlx5<br>Msx2	Inhibits terminal differentiation or cartilage resorption	[39]<br>[38]<br>[41]<br>[40]
Rbpj	Promotes terminal differentiation or cartilage resorption	[72]

Additional References

69. Arnold MA, Kim Y, Czubryt MP, Phan D, McAnally J, et al. (2007) MEF2C transcription factor controls chondrocyte hypertrophy and bone development. Dev Cell 12: 377–389. doi:10.1016/j.devcel.2007.02.004.

70. Mikasa M, Rokutanda S, Komori H, Ito K, Tsang YS, et al. (2010) Regulation of Tcf7 by Runx2 in chondrocyte maturation and proliferation. Journal of Bone and Mineral Metabolism 29: 291–299. doi:10.1007/s00774-010-0222-z.

71. Guo X, Zhao H (2012) Foxp1/2/4, New Transcriptional Regulators for the Chondrocyte Hypertrophy and Osteoblast Differentiation during Skeletal Ossification Minneapolis.

72. Kohn A, Dong Y, Mirando AJ, Jesse AM, Honjo T, et al. (2012) Cartilage-specific RBPj -dependent and -independent Notch signals regulate cartilage and bone development. Development 139: 1198–1212. doi:10.1242/dev.070649.
