# Supplementary material for: SHP2 Regulates Chondrocyte Terminal Differentiation, Growth Plate Architecture and Skeletal Cell Fates
Source: PLoS Genet. 2014 May 29;10(5):e1004364. doi: 10.1371/journal.pgen.1004364 (PMC4038465; doi:10.1371/journal.pgen.1004364)
Supplement: Table S5 — (PDF) [file pgen.1004364.s019.pdf]

Transcription factor	Effect on gene expression	Target gene	Reference
Msx2	positive	*Ihh*<br>*Alpl*<br>*Col10a1*	[40]
Mef2c	positive	*Col10a1*	[69]
Hey1	positive	*Col10a1*	[39]
Sox9	positive	*Col10a1*	[73]
	negative	*Spp1*<br>*Mmp13*	[38]
Egr1	positive	*Hpse*	[74]
		Spp1	[75]
	negative	*Acan*	[76]
Mafb	positive<br>negative	*Mmp13*<br>*Acan*	[77]
AP-1 complex	positive	*Mmp13*	[78]
		Mmp12	[79]
		Spp1	[80]
Rbpj	positive	*Mmp13*	[72]
Hmga1	positive	*Igfbp3*	[81]

Additional References

69. Arnold MA, Kim Y, Czubryt MP, Phan D, McAnally J, et al. (2007) MEF2C transcription factor controls chondrocyte hypertrophy and bone development. Dev Cell 12: 377–389. doi:10.1016/j.devcel.2007.02.004.

72. Kohn A, Dong Y, Mirando AJ, Jesse AM, Honjo T, et al. (2012) Cartilage-specific RBPj -dependent and -independent Notch signals regulate cartilage and bone development. Development 139: 1198–1212. doi:10.1242/dev.070649.

73. Dy P, Wang W, Bhattaram P, Wang Q, Wang L, et al. (2012) Sox9 Directs Hypertrophic Maturation and Blocks Osteoblast Differentiation of Growth Plate Chondrocytes. Developmental Cell 22: 597–609. doi:10.1016/j.devcel.2011.12.024.

74. de Mestre AM, Rao S, Hornby JR, Soe-Htwe T, Khachigian LM, et al. (2005) Early growth response gene 1 (EGR1) regulates heparanase gene transcription in tumor cells. J Biol Chem 280: 35136–35147. doi:10.1074/jbc.M503414200.

75. Liu Q-F, Yu H-W, Liu G-N (2009) Egr-1 upregulates OPN through direct binding to its promoter and OPN upregulates Egr-1 via the ERK pathway. Mol Cell Biochem 332: 77–84. doi:10.1007/s11010-009-0176-4.

76. Rockel JS, Bernier SM, Leask A (2009) Egr-1 inhibits the expression of extracellular matrix genes in chondrocytes by TNFalpha-induced MEK/ERK signalling. Arthritis Res Ther 11: R8. doi:10.1186/ar2595.

77. Zhang Y, Ross AC (2013) Retinoic acid and the transcription factor MafB act together and differentially to regulate aggrecan and matrix metalloproteinase gene expression in neonatal chondrocytes. Journal of Cellular Biochemistry 114: 471–479. doi:10.1002/jcb.24387.

78. Ahmad R, Sylvester J, Zafarullah M (2007) MyD88, IRAK1 and TRAF6 knockdown in human chondrocytes inhibits interleukin-1-induced matrix metalloproteinase-13 gene expression and promoter activity by impairing MAP kinase activation. Cell Signal 19: 2549–2557. doi:10.1016/j.cellsig.2007.08.013.

79. Wu L, Tanimoto A, Murata Y, Fan J, Sasaguri Y, et al. (2001) Induction of human matrix metalloproteinase-12 gene transcriptional activity by GM-CSF requires the AP-1 binding site in human U937 monocytic cells. Biochem Biophys Res Commun 285: 300–307. doi:10.1006/bbrc.2001.5161.

80. Sharma P, Kumar S, Kundu GC (2010) Transcriptional regulation of human osteopontin promoter by histone deacetylase inhibitor, trichostatin A in cervical cancer cells. Mol Cancer 9: 178. doi:10.1186/1476-4598-9-178.

81. Gasparini G, Gori MD, Paonessa F, Chiefari E, Brunetti A, et al. (2012) Functional relationship between high mobility group A1 (HMGA1) protein and insulin-like growth factor-binding protein 3 (IGFBP-3) in human chondrocytes. Arthritis Res Ther 14: R207. doi:10.1186/ar4045.
