# Supplementary material for: SHP2 Regulates Chondrocyte Terminal Differentiation, Growth Plate Architecture and Skeletal Cell Fates
Source: PLoS Genet. 2014 May 29;10(5):e1004364. doi: 10.1371/journal.pgen.1004364 (PMC4038465; doi:10.1371/journal.pgen.1004364)
Supplement: Table S6 — (PDF) [file pgen.1004364.s020.pdf]

**Transcript**	**Forward Primer**	**Reverse Primer**
Col9a3	AGGTACACCTTGAGGCCCTT	TCCCACTGGGGAACTAGGAG
Ihh	TCAAGGACGAGGAGAACACG	GAGTGATGGCCATCTTCATCC
Phospho1	CTGTTGGTCTCCAGCTGTCA	GCAGAAGCACATCATCCACA
Spp1	TGGCTATAGGATCTGGGTGC	TTGGCAGTGATTTGCTTTTG
Adamts5	GTGTCACATGAATGATGCCC	CGACCCTCAAGAACTTTTGC
Hmga2	TCTCCTGAGCAGGCTTCTTC	AAGGCAGCAAAAACAAGAGC
Egr1	GAGTCGTTTGGCTGGGATAA	CCTTCAATCCTCAAGGGGAG
Fosl1	CTCTTCCTCCTCTGGGCTG	TGGCCTATCCCCAGTACAGT
Actb	CAGCTTCTTTGCAGCTCCTT	AGGAGTCCTTCTGACCCATTC
